# Unraveling Alkali Ion Electron Donation for Enhancing Heterogeneous Catalytic Oxidation

**DOI:** 10.1002/advs.202514470

**Published:** 2025-10-08

**Authors:** Jin Wang, Zhenghui Zhang, Xianqiu Song, Rong Wang, Ying Xin, Yexin Zhang, Zhaoliang Zhang

**Affiliations:** ^1^ School of Chemistry and Chemical Engineering University of Jinan Jinan 250022 China; ^2^ Ningbo Institute of Materials Technology & Engineering Chinese Academy of Sciences 1219 Zhongguan West Road Ningbo 315201 China; ^3^ University of the Chinese Academy of Sciences 19A Yuquan Road Beijing 100049 China

**Keywords:** alkali promoter, catalytic oxidation, electron donation, single potassium ion, soot combustion

## Abstract

Alkali ions, widely employed as promoters, play crucial roles in heterogeneous catalysis. However, their electron donation mechanism remains poorly understood because alkali ions do not have transferable electrons as metallic alkalis do. Here, a new mechanistic pathway is identified for potassium (K) ions to act as electron donors by constructing single chains of K ions confined within hexagonal WO_3_ tunnels with theoretical limit‐breakthrough K concentrations. The as‐produced high‐electron‐density K^δ+^ (0<δ<1) ions not only facilitate the formation of oxygen vacancies that behave classical electronic effect but, more importantly, also directly donate electrons to [WO_6_] motif antibonding orbitals, thereby triggering the lattice oxygen activation. As a proof of concept, the resultant catalyst containing K^δ+^ ions predominantly promotes soot oxidation with O_2_, a challenging solid‐solid‐gas reaction in automotive catalysis, achieving a reaction rate 3.1‐fold greater than the conventional K⁺‐ion confined counterpart. The electron donation effect of metalloid K^δ+^ ions would be heuristic for enhancing other important heterogeneous catalytic reactions.

## Graphical abstract

A new mechanistic pathway for potassium (K) ions as electron donors is identified by constructing single K ion chains confined within hexagonal WO_3_ tunnels. The as‐produced high‐electron‐density K^δ+^ (0<δ<1) ions not only facilitate the formation of oxygen vacancies that behave classical electronic effect but, more importantly, also directly donate electrons to [WO_6_] motif antibonding orbitals, thereby triggering the lattice oxygen activation.

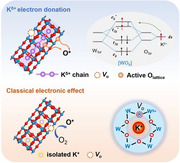

## 1 Introduction

Alkali metal promoters, highly reactive elements with an ease of losing a valence electron, have been extensively utilized in numerous key reactions, such as ammonia (NH_3_) synthesis,^[^ ^1^, ^2^ ^]^ Fischer‐Tropsch synthesis (FTS),^[^ ^3^ ^]^ as well as in the burgeoning field of CO_2_ capture and hydrogenation,^[^ ^4^, ^5^ ^]^ due to their remarkable ability to enhance the activity and/or stability of catalysts. Generally, they predominantly exist in ionic forms (e.g., salts or oxides) and are referred to act as either structural or electronic promoters. The former is primarily recognized for enhancing longevity by stabilizing catalytically active components or intermediates.^[^ ^6^, ^7^ ^]^ However, the role of the latter as a key factor in reaction acceleration remains a topic of ongoing scientific inquiry, since the introduction of the alkali promotion effect in 1845^[^ ^8^, ^9^ ^]^ (Illustration S1, Supporting Information).

The canonical understanding held that alkali ions could modify the apparent electronic state of active components to enhance the adsorption and activation of reactant molecules, thereby accelerating catalytic reaction kinetics.^[^ ^10^, ^11^ ^]^ However, it appears theoretically impracticable that closed‐shell alkali cations with no transferable *s* electrons (electronic configuration, ns^0^) donate electrons to electron acceptors, such as active metal sites or adsorbed reactant molecules. This contention is supported by ultrahigh vacuum studies on model Fe (100) single‐crystal catalysts, which reveal no detectable electronic promotion of KO*_x_* species on the ammonia synthesis rate, while a Fe catalyst with metallic K promoter would work better^.[^ ^12^, ^13^ ^]^ In the light of the ongoing debate, a number of researchers are attempting to reconcile these observations. de Jong's group suggested a synergistic effect of Na (alkali cation) plus S (counterion), which jointly act as electron donors to improve the specific selectivity of Co‐based catalysts.^[^ ^14^ ^]^ Alternatively, the Zheng group^[^ ^15^ ^]^ proposed the columbic attraction of alkali cations (Na^+^) to stabilize negatively charged intermediates and transition states, thereby promoting the catalytic hydrogenation of atomically dispersed Ru(III) on Al_2_O_3_ catalysts. Although these findings present remarkable insights into understanding the alkali promotion effects, the question as to whether and how the alkali ions to donate electrons to facilitate catalyst performance remains ambiguous.

To address the above electron donation issues, the rational modulation of the electronic structure of alkali ion promoters is of crucial importance. However, it is a considerable challenge, as alkali metals inherently exhibit strong tendencies to lose their single valence electrons. Recent studies have demonstrated that alkali species could exhibit distinct electron donation capabilities depending on their chemical compositions (metallic, hydride, or oxide/salt forms), leading to varying promotional effects on catalytic activity.^[^ ^2^, ^13^ ^]^ Owing to the structural and compositional diversity of these alkali promoters, precisely clarifying the electronic effects of alkali ions remains highly problematic. Fortunately, a strategy has been reported to modulate transition metal electronic states through confinement effects of zeolite cages^[^ ^16^ ^]^ and oxide tunnels.^[^ ^17^ ^]^ Although these approaches have been employed to confine alkali ions, only isolated monovalent alkali ions have been formed in such systems.^[^ ^18^, ^19^ ^]^ Consequently, the electron donation phenomena of alkali ions have yet to be observed, let alone elucidating their electron donation mechanism.

Herein, we constructed a K‐promoted catalyst featuring K ions assembling into chains within the hexagonal phase tungsten (W) trioxide (HWO) tunnels. We chose K as the alkali model because it is the most frequently employed promoter in heterogeneous catalysis. Harnessing the confinement effects of the HWO framework structure, the distinct electronic state of K ions from conventional K^+^ ions was achieved through controlling K precursor feeding using a high‐temperature‐driven ion diffusion‒coordination strategy. Experiments combined with density function theory (DFT) calculations validated the creation of high electron density K^δ+^ (0 < δ < 1) ions, which can directly donate electrons to [WO_6_] motif antibonding orbitals, activating strong W─O bonds, thereby inducing the formation of reactive oxygen species (O^*^), a critical intermediate in heterogeneous catalytic oxidation reactions. To exemplify this innovative electron donation effect, catalytic soot oxidation, a challenging solid (soot)‐solid (catalyst)‐gas (O_2_) reaction in automotive catalysis, was applied as a probe reaction. The K^δ+^‐confined catalyst yields a reaction rate 3.1‐fold greater than the K⁺‐confined counterpart. The elucidation of the electron donation mechanism of alkali‐metal ions, a long‐plagued enigma in the catalytic community, represents a significant advancement for the rational design of catalysts with alkali ion promoters.

## 2 Results and Discussion

### 2.1 Catalyst Design, Structural Characterizations and K Ion Electronic States

**Figure 1:**
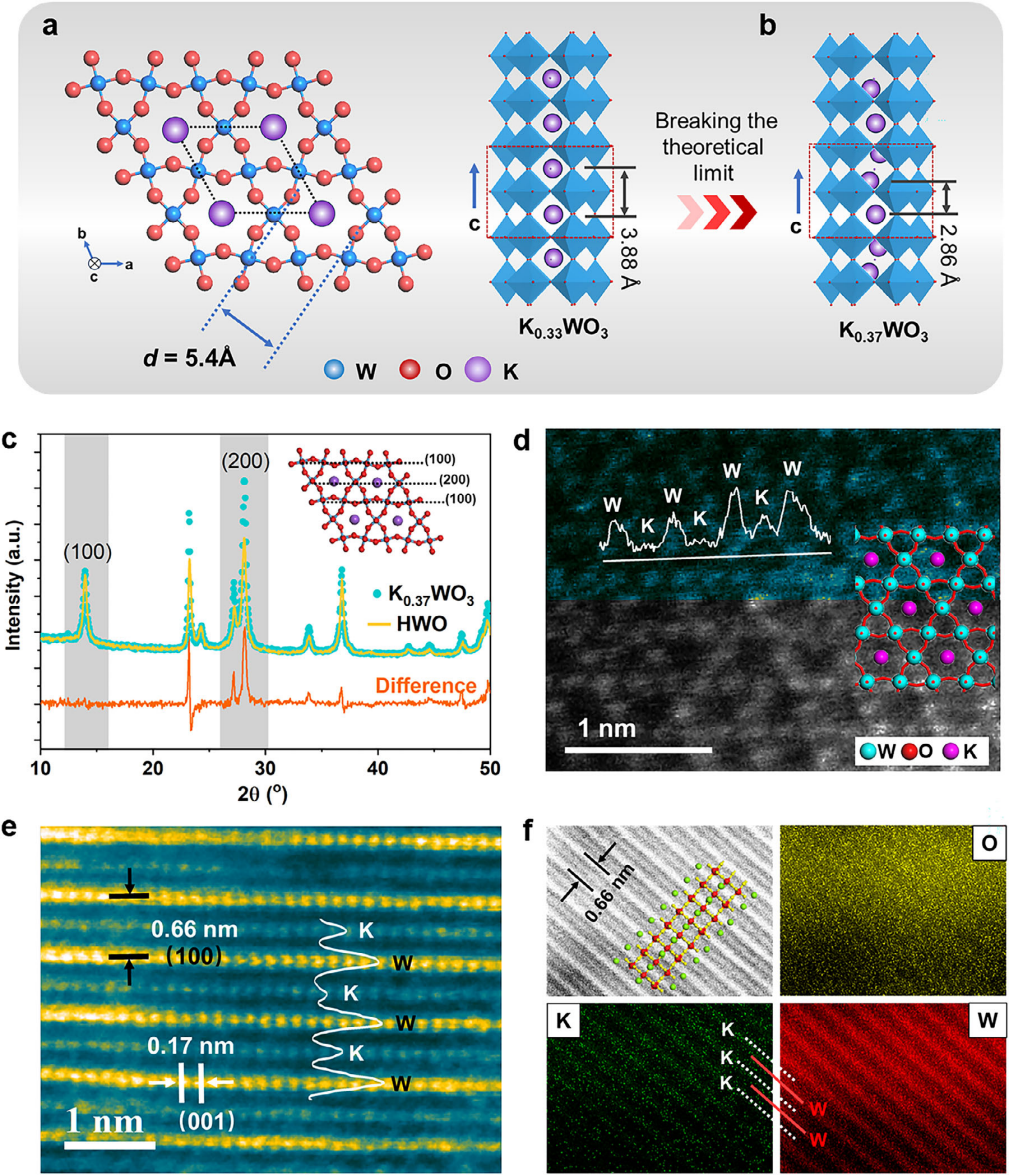
Catalyst design and structural characterizations of K_0.37_WO_3_. a) Confined K_12c_ atoms into the hexagonal phase tungsten trioxide tunnel (K_0.33_WO_3_) in the a‐b plane (left) and along the *c*‐axis (right), showing the tuning principle. b) K_0.37_WO_3_ structure along the *c*‐axis. The value (3.88 and 2.86Å) corresponds to the distance between K atoms. c) Synchrotron X‐ray diffraction (SXRD) patterns of K_0.37_WO_3_ and HWO, and their difference (orange line). The inset shows the side view of (100) and (200) planes. d) HAADF‐STEM image of K_0.37_WO_3_ projected along [001], with the corresponding atomic model and intensity variations along the white line. e) HAADF‐STEM image of K_0.37_WO_3_ projected along [‐120], the lattice spacing, and the contrast intensity variations along the white line. f) HAADF‐STEM image with a representative atomic model and corresponding elemental mapping of O, K, and W. The yellow, green, and red spheres represent O, K, and W atoms, respectively.

According to the principle of confinement effect and the properties of K, both hollandite‐type α‐MnO_2_ and hexagonal tungsten trioxide (HWO) could act as hosts for alkali cations. However, the alkali promoter effect is largely overshadowed by the high inherent redox activity of α‐MnO_2_.^[^ ^20^ ^]^ Due to the strong W‐O interaction, i.e., chemically inactive, the reactivity of [WO_6_] motifs relies on additional components, such as alkali ion promoters. Furthermore, the optimal tunnel size (5.4 Å) of HWO allows the accommodation of K^+^ ions,^[^ ^18^ ^]^ thereby enabling precise control over their concentration within the tunnels (**Figure** **1**a). According to the density function theory (DFT) simulations and previous literature,^[^ ^21^, ^22^ ^]^ K ion can easily migrate from the surface into and locate within the HWO tunnels, with barrier energies of 0.18 eV, and the migration process is thermodynamically spontaneous (Figure S1, Supporting Information). Moreover, confined K ions preferentially occupy the K_12c_ sites, where each K ion is coordinated by twelve oxygen atoms on the walls of hexagonal‐prism tunnels (Figure S2, Supporting Information). The complete filling of K_12c_ sites would result in the formation of K_0.33_WO_3_, where 0.33 is identified as a theoretical limit value of *x* in K*_x_* WO_3_ when the atomic ratio of W is set to 1 in previous literature.^[^ ^21^, ^22^ ^]^ Interestingly, our DFT simulations verified that K_6c_ (K ions coordinated by six oxygen atoms) is also a feasible K coordination structure, the stability of which is slightly weaker than K_12c_ (Figure S2 and Note S1, Supporting Information). As such, when the K content in K*_x_* WO_3_ exceeds the theoretical limit (i.e., *x* > 0.33), a closer K‐K distance would promote the formation of partial K_6c_ configurations (Figure 1b), thereby enhancing ion mobility and potentially inducing a unique electronic state.

Therefore, a high‐temperature‐driven ion diffusion‒coordination strategy was developed (Figure S3, Supporting Information), involving a two‐stage temperature‐programmed process. In the initial stage, a relatively low temperature (350 °C) is applied to facilitate the diffusion and insertion of K^+^ ions into the tunnels of HWO, thereby leading to the formation of K_12c_. Subsequently, a higher temperature (500 °C) enabled the incorporation of K^+^ beyond the theoretical limit, resulting in the formation of a partial metastable K‐O coordination structure (K_6c_) in K_0.37_WO_3_. In the conventional one‐pot solid‐phase synthesis of K*_x_* WO_3_, typically, high‐temperature treatment was also employed. Owing to the concurrent assembly of the W–O framework and the confined K ions, together with the use of a stoichiometric amount of potassium, high‐level insertion of alkali ions was not achieved.^[^ ^21^, ^23^ ^]^ Using this innovative method, the highest achievable K concentration confined K catalyst (K_0.37_WO_3_), breaking through the theoretical limit, was successfully synthesized. To eliminate any surface K^+^ species, the synthesized K_0.37_WO_3_ catalyst was washed with ultrapure water under vigorous stirring for 24 h. The structural formula was determined by means of elemental analysis via inductively coupled plasma atomic emission spectrometry (ICP‒OES) (Table S1, Supporting Information). The synthesis mechanism is delineated in Note S2 (Supporting Information).

In order to verify the exact positions and chemical states of the trapped K ions, the structures of the as‐synthesized catalysts were characterized in detail. High‐resolution transmission electron microscopy (HRTEM) revealed that the rod‐shaped K_0.37_WO_3_ grows along the [001] direction, with two (001) top facets and six (100)/(1–10) side facets. The tunnel axes are parallel to the [001] direction of the nanorod (Figure S4, Supporting Information). Moreover, highly dispersed K species are distributed throughout the WO_3_ nanorod in the scanning electron microscopy (SEM) images and elemental mapping (Figure S5, Supporting Information).

The synchrotron X‐ray diffraction (SXRD) patterns show that the K_0.37_WO_3_ exhibits a hexagonal crystal structure and ordered K ions occupy the centre of the hexagonal tunnels, which is featured by a strong enhancement of the (200) peak by normalizing the intensity of the (100) peak (Figure 1c Orange difference line). Rietveld refinement (Figure S6 and Table S2, Supporting Information) indicates that the (002) superposition results from K ions on the (002) plane, confirming their dominant occupancy at the Wyckoff 1a sites. Direct observation of the precise locations of K ions in K_0.37_WO_3_ is realized using scanning transmission electron microscopy equipped with a spherical aberration‐corrected high‐angle angular dark field detector (HAADF‐STEM). The down‐tunnel view image (Figure 1d) shows that K atoms occupy the central positions of the hexagonal tunnels formed by tungsten atomic columns, as further confirmed by the STEM image (Figure 1e) and the corresponding energy‐dispersive spectroscopy (EDS) elemental mapping (Figure 1f), which clearly show the alternating arrangement of K and W columns along the [100] crystallographic direction. These results conclusively confirm the successful synthesis of the K_0.37_WO_3_ catalyst, where single K ion chains are confined in the HWO tunnels by insertion of K contents exceeding the theoretical limit.

To highlight specific properties of the synthesized K_0.37_WO_3_ catalyst, two other K*_x_* WO_3_ catalysts, K_0.32_WO_3_ (approaching the theoretical *x* value) and K_0.29_WO_3_ (below the theoretical *x* value), were synthesized using the aforementioned synthesis strategy (Figure S3, Supporting Information). In addition, a KNO_3_‐impregnated sample (K/HWO) with comparable K content to K_0.37_WO_3_ was prepared by drying without calcination, yielding only surface‐adsorbed K^+^ species. These carefully designed catalysts enable us to provide a basis for understanding the differences in structural and electronic properties. Comprehensive structural characterizations confirm the successful synthesis of K_0.29_WO_3_ and K_0.32_WO_3_ catalysts (Figures S7 and S8 and Table S2, Supporting Information).

**Figure 2:**
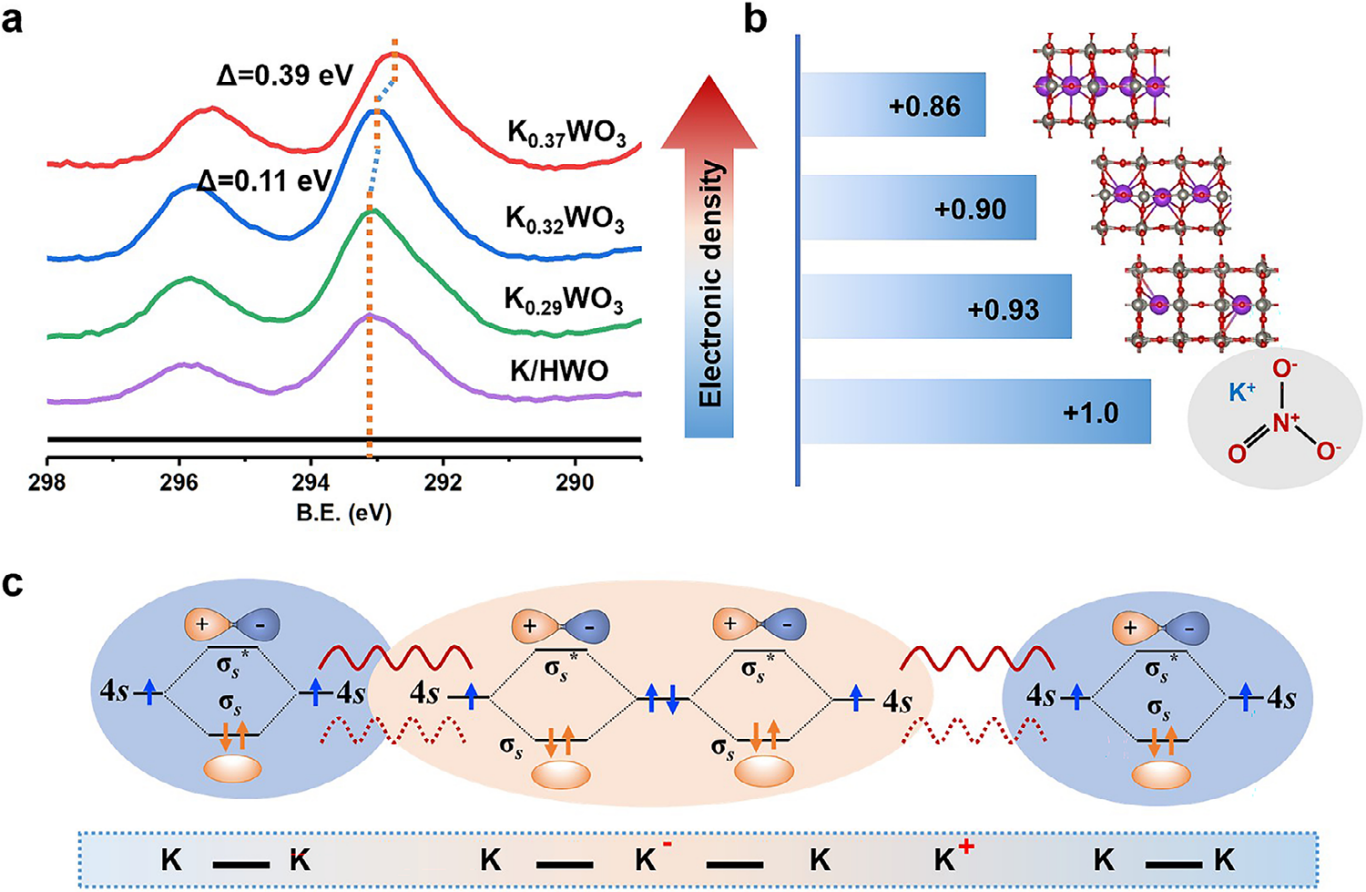
Electronic states of K*_x_* WO_3_ (*x* = 0.29, 0.32, and 0.37). a) K 2p XPS results for K/HWO, K_0.29_WO_3_, K_0.32_WO_3_, and K_0.37_WO_3_, b) Bader charge analysis of K cations within the corresponding models based on DFT calculations. c) Molecular orbital energy level diagram for the K chain resonance model. In the blue area, two adjacent K atoms donate their valence electrons (4s electrons) to form a localized covalent bond, while in the orange area, the electron transfer results in the formation of discrete K^+^ ions with 4s electrons being delocalized to create a K^−^ center, thereby establishing two covalent bonds sharing a single K^−^ ion. The wavy lines represent the resonance between two potential electronic configurations.

To acquire the electronic state of K ions in K*_x_* WO_3_ (*x* = 0.29, 0.32, and 0.37), X‐ray photoelectron spectroscopy (XPS) was carried out. A clear peak downshift of 0.39 eV was observed for K_0.37_WO_3_ in comparison with K/HWO, while only 0.11 eV and negligible shifts were detected for K_0.32_WO_3_ and K_0.29_WO_3_, respectively, indicating higher electron density around K ions in K_0.37_WO_3_ (**Figure** **2**a), designated as K^δ^⁺ (0 < δ < 1). The variations in the electron state of the K ions were further confirmed by K K‐edge X‐ray absorption near‐edge structure (XANES) spectra (Figure S9, Supporting Information). The features of the spectra for K*_x_* WO_3_ (*x* = 0.29, 0.32, and 0.37) are notably differ from those of the KNO_3_ precursor, with an enhanced white line peak centered at ≈3616 eV and a shoulder at 3621.4 eV, indicating that K ions are inserted into the nanochannel, which is consistent with the previous report.^[^ ^24^ ^]^ The redshift of white line features (band B) for K_0.37_WO_3_ (at 3616.1 eV) compared to that of K_0.29_WO_3_ (at 3616.4 eV) and K_0.32_WO_3_ (at 3616.3 eV) suggests the valence state reduction of K ions in K_0.37_WO_3_, i.e., the emergence of K^δ^⁺ species (Figure S10, Supporting Information). As identified by XPS and XANES results, two kinds of electronically tunable K‐based catalysts (K^δ^⁺ in K_0.37_WO_3_ and K⁺ in K_0.32_WO_3_ and K_0.29_WO_3_) were constructed by regulating the K content. The variations of the electron state of K ions within K*_x_* WO_3_ are likely associated with the appearance of K_6c_ species, thereby shortening the K–K distance and leading to the plausible interaction.

For testifying above analysis, three representative theoretical models are constructed for K_0.29_WO_3_, K_0.32_WO_3_, and K_0.37_WO_3_, denoted as 1K‐HWO, 2K‐HWO, and 3K‐HWO, respectively (Figure S11, Supporting Information) to elucidate the alterations in the electron density of K ions within K*_x_* WO_3_. As shown in Figure 2b, the calculated Bader charge of the K species decreases from +0.93 (1K‐HWO) to +0.90 (2K‐HWO) and +0.85 (3K‐HWO), which are in close agreement with the XPS results. Specifically, the appearance of K_6c_ for K_0.37_WO_3_ enables close contact between K ions, facilitating metalloid K–K interactions and thus allowing these ions to retain some of their valence electrons to form K*^δ^* ^+^ (0 < *δ *< 1) (Figure S12, Supporting Information), similar to Ag–Ag bonds (Ag^+0.82^) in the tunnels of the hollandite‐type α‐MnO_2_ rods.^[^ ^17^ ^]^ In contrast, the K ions (K_12c_) in the K_0.29_WO_3_ and K_0.32_WO_3_ systems are spatially isolated and do not come into close enough contact to form K‒K bonds, therefore predominantly existing in the form of K^+^ ionic states.

The nature of K‒K bonds within linear K ion chain arrays was corroborated according to fundamental principles of metallic bonding, outlined by Linus Pauling.^[^ ^25^ ^]^ As illustrated in Figure 2c, two elementary K‐K configurations exist, and the unsynchronized resonance between them creates the long‐range interactions among linearly arranged K atoms. The formation of K‒K interactions effectively reduces the energy of the system by mitigating the repulsion between K ions. Consequently, the emergence of K‒K bonds not only facilitates the development of electronically tunable K‐based catalysts but also enables the stabilization of K ions, as for metastable K_6c_ configurations, which provide an ideal platform for elucidating the fundamental electron donation mechanism.

### 2.2 Spectral Characterizations of Electron‐Occupied Molecular Orbitals

**Figure 3:**
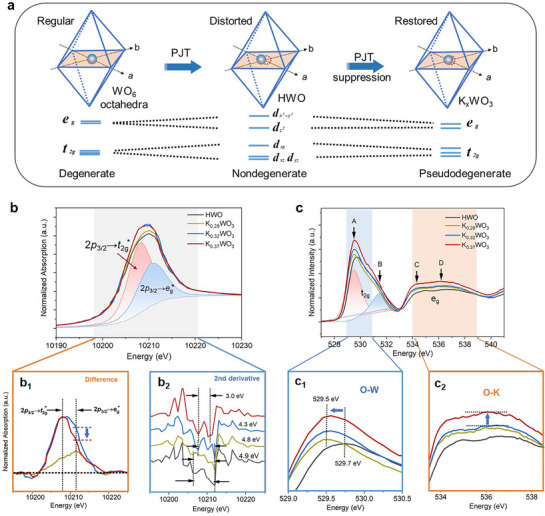
Identification of the electron donation process and mechanism. a) Changes in the unit cell structure affected by the pseudo Jahn–Teller effect, and the corresponding degenerate and nondegenerate energy levels. b) W *L_3_*‐edge XANES spectra and the corresponding (b_1_) differential XANES spectra of K*_x_* WO_3_ with respect to that of HWO (black dotted line) and (b_2_) second derivative spectra and the corresponding splitting energy for K*_x_* WO_3_ and HWO. c) Oxygen K‐edge XANES spectra and the corresponding partial enlarged view (c_1_) O‐W interaction and (c_2_) O‐K interaction.

To elucidate the capacity of K^δ+^ to donate electrons, precise structural changes of the WO_3_ framework were first analysed, as the electron donation is concomitant with lattice distortion, interpreted as pseudo Jahn–Teller (PJT) effect, which is encountered in many systems of solid‐state crystals, especially in octahedral metal complexes.^[^ ^26^, ^27^, ^28^ ^]^ According to SXRD (Figure S13, Supporting Information) and Rietveld refinement (Table S2, Supporting Information) analysis, the pronounced lattice distortion was observed in K_0.37_WO_3_ with the elongation of the W─O bonds along the *c*‐axis and contraction of these bonds along the *a*/*b* axis compared to HWO, which was further evidenced by Raman spectra (Figure S14, Supporting Information). DFT calculations verified the lattice distortion derived from the displacement of W^6+^ in the *a‒b* plane of oxide octahedra (Figure S15, Supporting Information). For HWO, the off‐centre displacement of W^6+^ (PJT distortion) has the effects of removing the orbital degeneracy and lowering the overall energy (**Figure** **3**a). With the insertion of K ions, however, a noticeable reduction in the deviation of the W─O bond lengths in the *a‒b* plane is observed in K*_x_* WO_3_ (Figure S15, Supporting Information), indicating the distorted WO_6_ octahedra increasingly approached a regular octahedral structure, i.e., PJT distortion suppression accompanied by the increased degeneracy of energy levels (Figure 3a). This particular degeneracy is different from the conventional quantum degeneracy, while it stems from the asymmetry of the electron distributions, referred to as pseudodegeneracy.^[^ ^26^, ^29^ ^]^ In particular, the strongest suppression of the PJT distortion was observed for K_0.37_WO_3_, which would be strongly related to specific electron configurations compared to other K*_x_* WO_3_ samples.

Accordingly, the spectral characterizations coupled with molecular orbital (MO) distributions were carried out to probe energy level electron distributions. In the HWO, the ground state configuration shows that electrons fully occupy the nonbonding orbitals, while the *t*_2g_^*^ and *e*_g_^*^ antibonding orbitals remain empty (Figure S16, Supporting Information). In the W *L_3_*‐edge XANES spectra (Figure 3b), two deconvoluted peaks corresponding to electron transitions from W 2p_3/2_ to the split *t*_2g_^*^ and *e*_g_^*^ states^[^ ^30^ ^]^ emerged in both HWO and K*_x_* WO_3_. However, in the differential XANES spectra of K*_x_* WO_3_ with respect to HWO (Figure 3b1), a remarkable weakening of the *e*_g_^*^ peak, accompanied by the negligible difference of the *t*_2g_^*^ peak for K_0.37_WO_3_ relative to K_0.32_WO_3_ reveals the partially occupied *p‐d e*_g_^*^ antibonding orbitals^[^ ^31^, ^32^ ^]^(for more explanations, see Figure S17 and Note S3, Supporting Information). This result provides direct evidence that electrons from K^δ+^ flow into the high‐energy *e*_g_^*^ orbitals of the [WO_6_] motifs in the case of K_0.37_WO_3_. Furthermore, the corresponding second‐derivative spectra (Figure 3b2) demonstrated that the apparent electron filling in the *e*_g_^*^ orbital for K_0.37_WO_3_ benefits from the strong mixing between the *t*_2g_^*^‐*e*_g_^*^ orbitals due to the smallest splitting energy (3.0 eV), which is considerably lower than those of HWO (4.9 eV), K_0.29_WO_3_ (4.8 eV) and K_0.32_WO_3_ (4.3 eV). This decrease in splitting energy not only reflects the pseudodegenerate W 5d orbitals but also provides evidence for the feasibility of the electron donation to the *e*_g_^*^ orbital despite its higher energy level compared with that of the *t*_2g_^*^ orbital.^[^ ^33^ ^]^

The electron transition to the *e*_g_^*^ orbital was further confirmed by the O K‐edge XANES spectra. As shown in Figure 3c, the first intense peak (≈530 eV) corresponds to the hybridization between the oxygen 2p states and W 5d*t*_2g_ states, where peak A is attributed to the *t*_2g_^*^ conduction band and the shoulder‐peak B reflects the anisotropy of *t*_2g_^*^.^[^ ^34^, ^35^ ^]^ After K ion insertion, a notable down‐shift of peak‐A in energy (ΔE = 0.2 eV) is observed, indicating a reduction in the energy of the unoccupied *t*_2g_^*^orbitals for K*_x_* WO_3_ (Figure 3c1). According to DFT calculations, the confinement of K ions induces a downshift of the lowest unoccupied molecular orbital (LUMO) state (Figure S18, Supporting Information), resulting in band‐tail states below the Fermi level, which can be attributed to the conduction of K electrons through the hybridized W_t2g*_ and oxygen π^*^ orbital pathways.^[^ ^36^, ^37^ ^]^

Based on the above discussion, the insertion of K ions causes a lowering of orbital energy levels, thereby enhancing the propensity for electron transfer. Furthermore, a decrease in orbital splitting energy, as observed in Figure 3b2, promotes strong *t*_2g_^*^‐*e*_g_^*^ orbital mixing (Figure S19, Supporting Information). Consequently, electrons from K^δ+^ ion chains can populate the *e*_g_^*^ orbitals of the [WO_6_] motifs. This unique electronic configuration acts as the fundamental driving force for electron donation behind the pronounced suppression of PJT distortion described above.

### 2.3 Distinguishing Electron Donation of K^δ+^ Ions from Conventional Oxygen Vacancies

**Figure 4:**
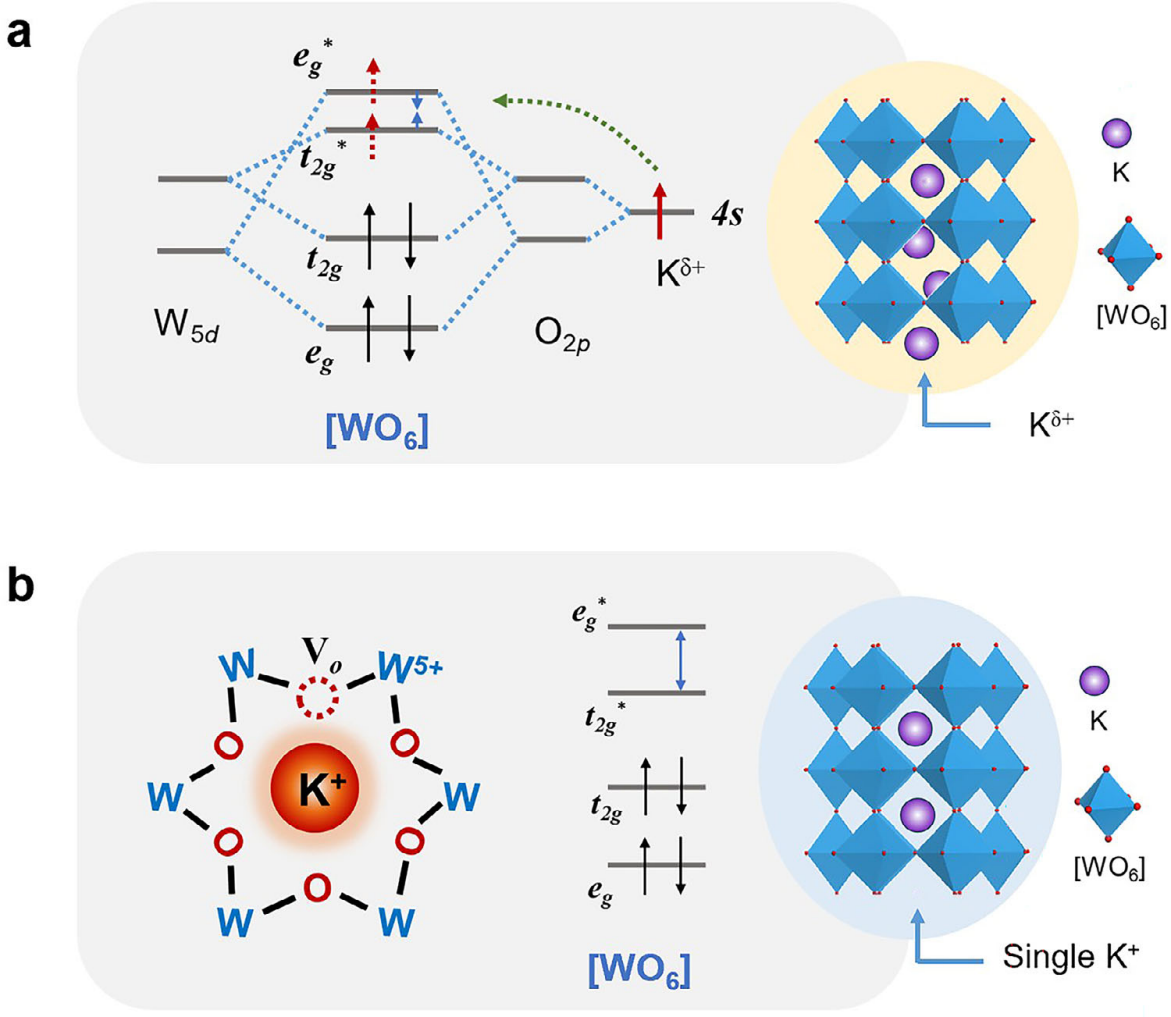
Schematic representation of the electron donation mechanism. a) Electron‐donation mechanistic pathway from high‐electron‐density K^δ+^ ions to [WO_6_] motif antibonding orbitals in K_0.37_WO_3_ and b) Classical electron effects for the formation of V_o_ or W^5+^ in K_0.29_WO_3_ and K_0.32_WO_3_.

To further explore the contributions of K^δ+^ ions to the electron population in the *e*_g_^*^ orbitals, the K‐O interaction was analysed based on the O K‐edge XANES spectra. As observed in Figure 3c2, the broad peaks at 534–538 eV (peaks C and D) correspond to the hybridization of the O 2p states and W 5d‐*e*_g_ orbitals, which are significantly influenced by the O(2p)‐K(4s) interaction.^[^ ^34^, ^38^ ^]^ The drastic increase in peak intensity from K_0.29_WO_3_ and K_0.32_WO_3_ to K_0.37_WO_3_ is attributed to the increase in the number of O 2p empty orbitals, i.e., the presence of a partially covalent rather than complete ionic K─O bond in K_0.37_WO_3_ (or electron sharing between the K and O atoms).^[^ ^34^ ^]^ The increased K‐O covalency in K_0.37_WO_3_ is further supported by the calculated electronic localization function (ELF), which shows a more uniform electron distribution with the highest electron density (average ELF = 0.64) in the region between the K and O atoms (Figure S20, Supporting Information). This enhanced K‐O covalency is attributed to the emergence of electron‐rich K^δ+^ ions, which facilitate electron sharing with surrounding O atoms. The metalloid electron donation behavior of these K^δ+^ ions perturbs the initial electron distribution within the WO_3_ framework, ultimately resulting in the unique electronic configuration as observed in K_0.37_WO_3_ (**Figure** **4**a).

However, in previous theoretical studies, oxygen vacancy (V_o_) was usually suggested to act as the electron source to donate electrons.^[^ ^39^, ^40^ ^]^ To distinguish this from the above K^δ+^ ions, electron paramagnetic resonance (EPR) spectra and O 2*p* XPS spectra were employed to characterize the V_o_ in K*_x_* WO_3_. The EPR spectra show a nearly linear increase in V_o_ concentration with increasing K contents (Figure S21, Supporting Information), which is consistent with the V_o_‐related oxygen signal (i.e., O_II_, active oxygen) observed in XPS spectra (Figure S22, Supporting Information), with the order K_0.29_WO_3_ < K_0.32_WO_3_ < K_0.37_WO_3_. Therefore, the oxygen deficiency in K*_x_* WO_3_ arises from K‐ion doping, as the incorporation of low‐valent K ions disrupts the charge neutrality of the initial WO_3_ lattice, triggering the generation of W^5+^ species and concomitant V_o_, as supported by W *4f* XPS analysis (Figure S23, Supporting Information). The V_o_ is responsible for the electron traps, localizing electrons at the defect sites or metal sites, thereby affecting the chemical properties of materials.^[^ ^41^ ^]^ This regulating role of alkali ions on the chemical states of oxides was traditionally considered as the alkali ion electronic promotion effect, as observed in K_0.29_WO_3_ and K_0.32_WO_3_ (Figure 4b), which might incur the progressive suppression of PJT distortion from K_0.29_WO_3_ to K_0.32_WO_3_. However, the observed correlation between V_o_ and structural distortion does not provide a sufficient explanation for the unique electronic characteristics of K_0.37_WO_3_, such as apparent electron filling in *e*_g_^*^ orbitals and partial covalency between K and O, as discussed above, which exclusively supports the electron donation pathway from electron‐rich K*^δ^* ^+^ species to antibonding orbitals (*e*_g_^*^) and the resultant strongest suppression of PJT distortion as unravelled in K_0.37_WO_3_.

### 2.4 Donation Effects on Activation of Lattice Oxygen

**Figure 5:**
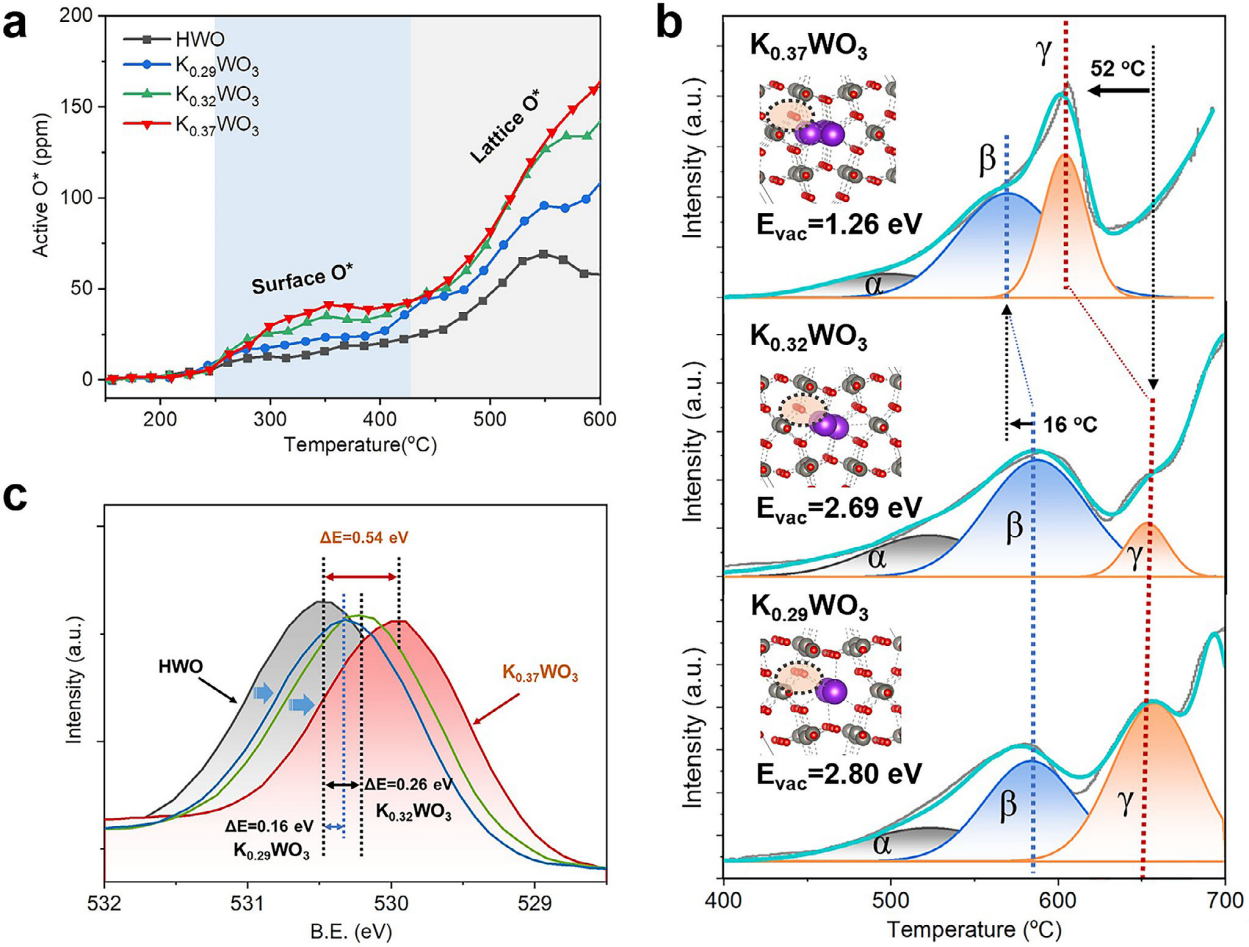
Activity of lattice oxygen. a) Soot‐TPR results for K*_x_* WO_3_ (*x* = 0.29, 0.32, and 0.37). b) H_2_‐TPR results for K*_x_* WO_3_, where the three peaks are attributed to the strong adsorption oxygen species (α), surface lattice oxygen (β), and bulk lattice oxygen (γ). Inset: Formation energy of oxygen vacancies and the corresponding oxygen atoms in K*_x_* WO_3_ models based on DFT calculations. c) O 1s XPS results and O_I_ peak shift analysis for K*_x_* WO_3_ compared to HWO. The ΔE values represent the shifts in the binding energy.

To elucidate the electron donation effect of the K^δ+^ ions, the activation of lattice oxygen was first investigated by temperature‐programmed reduction with soot (soot‐TPR) and H_2_ (H_2_‐TPR), which is directly related to activity in heterogeneous catalytic oxidation processes via the Mars‐van Krevelen (MvK) mechanism. As shown in **Figure** **5**a (soot‐TPR), K_0.37_WO_3_ exhibits the highest concentrations of activated surface and bulk lattice O^2−^ in the three K*_x_* WO_3_ samples. In the H_2_‐TPR spectra (Figure 5b), the reduced peaks of surface lattice oxygen (peak β) and bulk lattice oxygen (peak γ) both shift markedly to lower temperatures compared to those for K_0.32_WO_3_ and K_0.29_WO_3_, suggesting higher activity of lattice O^2−^ in K_0.37_WO_3_. Analogously, DFT calculations show that the formation energy of oxygen vacancies (*E*_vac_) in the K_0.37_WO_3_ model decreases sharply to only 1.26 eV, which is less than half the *E*_vac_ in the K_0.32_WO_3_ (2.69 eV) and K_0.29_WO_3_ (2.80 eV) models, as shown in the inset of Figure 5b. These results directly confirm the notable activation of lattice oxygen in K_0.37_WO_3_ due to the confined K^δ+^ doping. This enhanced activation of lattice oxygen atoms should be ascribed to the electron filling of antibonding orbitals (especially e_g_^*^), reducing the bond order and bond energy of M─O (metal‐oxygen) bonds, and thereby boosting the reactivity of lattice O^2−^ species.^[^ ^42^ ^]^ Subsequently, projected crystal orbital Hamilton population (pCOHP) calculations were performed to evaluate the chemical bonding strength of the W‐O interaction. In contrast to HWO, 1K‐HWO, and 2K‐HWO, the K^δ+^ ions in the WO_3_ tunnel significantly weaken the W‐O bonding in 3K‐HWO, as evidenced by the presence of occupied antibonding states below the Fermi level and the lowest integrated value of ‐pCOHP (‐IpCOHP) for W‐O in 3K‐HWO (Figure S24, Supporting Information).

Furthermore, O 1s XPS spectra (Figure 5c) show a pronounced downshift (ΔE = 0.54 eV) of the O_I_ peak (assigned to the surface lattice oxygen, O^2−^) in K_0.37_WO_3_ compared to HWO, suggesting the presence of the high‐electron‐density lattice oxygen in K_0.37_WO_3_ induced by K^δ+^ electron donation. In contrast, the shifts are significantly small for K_0.29_WO_3_ (0.16 eV) and for K_0.32_WO_3_(0.26 eV), highlighting the critical role of the K^δ+^ species in facilitating the W─O bond activation. The above electron donation‐induced lattice oxygen release is consistent with our previous finding that electrons pass through conductive oxide catalysts, such as antimony‐doped tin oxide (ATO), can drive the activation of lattice oxygen, which is responsible for soot ignition below 75 °C.^[^ ^43^ ^]^ Interestingly, in the present case, the electrons occupying the antibonding orbitals of the metal‐oxygen MOs are derived from the confined high‐electron‐density K^δ+^ chains instead of from external currents. Although the electron load is substantially lower than the externally applied current input, this study validates the feasibility of boosting electron‐donating capability through rational catalyst design.

### 2.5 Performances for Catalytic Soot Combustion

**Figure 6:**
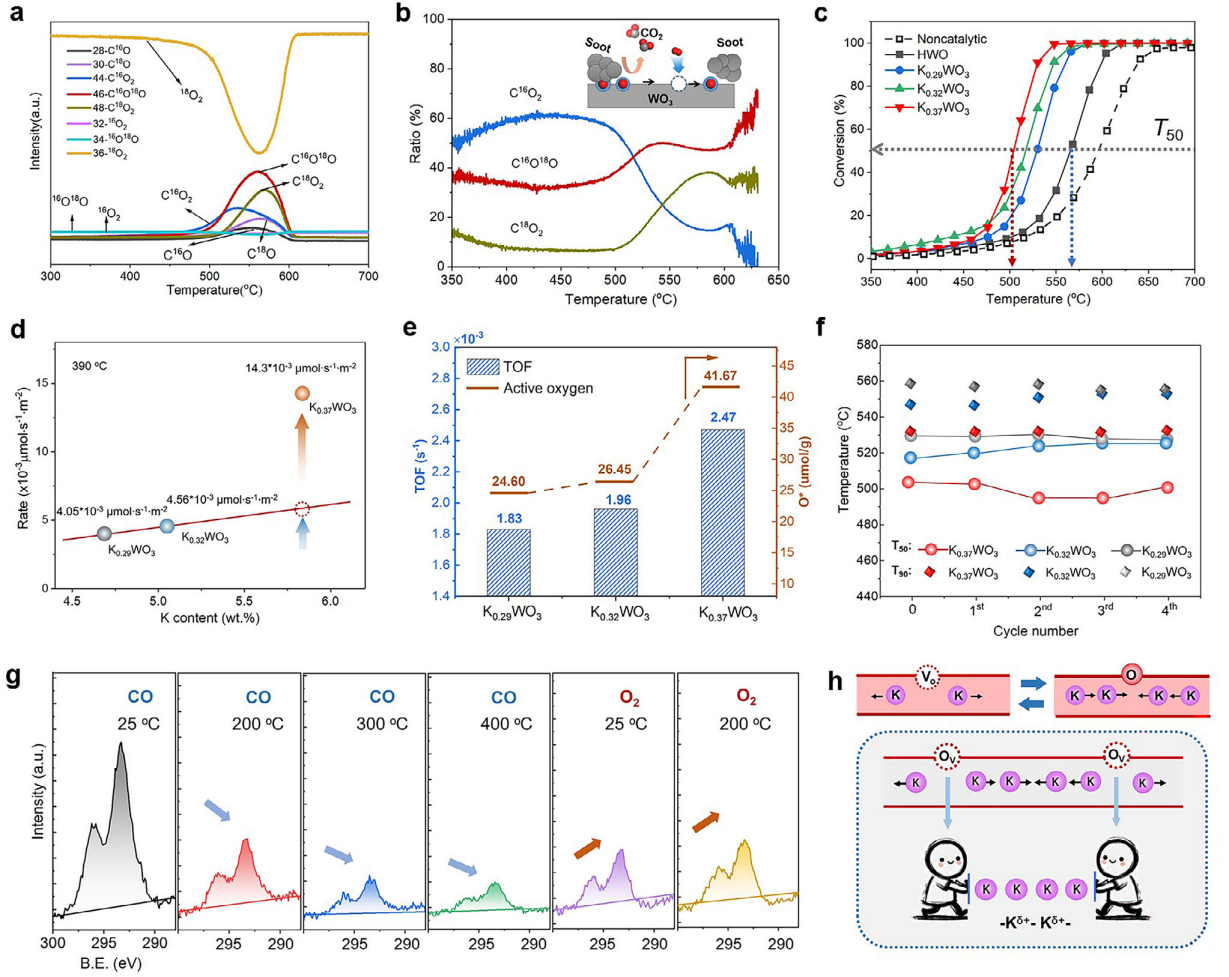
Comparison of catalytic performance of K*_x_* WO_3_ catalysts in soot combustion. a) Temperature‐programmed oxidation (TPO) of soot with 1% ^18^O_2_ isotopic in He for K_0.37_WO_3_ and b) variation in the CO_2_ production ratio as a function of temperature. The inset shows the Mars–van Krevelen mechanism for catalytic soot combustion. c) Soot conversion under the tight contact condition between catalyst and soot using 5 vol.% O_2_ in He. d) Isothermal reaction rate at 390 °C. Solid circles represent experimentally measured values, while dashed circles represent predicted values based on a linear correlation between V_o_ concentration (directly related to K^+^ content) and the amount of active oxygen species generated by O_2_ activation. e) Active oxygen content and TOF values for K_x_WO_3_ samples using soot as a probe molecule, determined via the isothermal anaerobic titration method. f) Cycling stability of K*_x_* WO_3_ for soot combustion in 5 vol.% O_2_/He. g) Evolution of K 2p peak intensity in the NAP‐XPS spectra for K_0.37_WO_3_ catalyst (from left to right), which was first exposed to CO at different temperatures and then to O_2_. h) The migration of K ions with the alternation between lattice oxygen and V_o_ at sites (up), and the mechanism of K‐K interaction mediated by V_o_ (down).

The challenging solid (soot)‒solid (catalyst)‒gas (O_2_) triphase reaction involved in soot combustion for automobile catalysis was employed as a probe reaction to investigate the catalytic performance, since it strongly depends on the high activity and mobility of active oxygen (O^*^) due to diffusion limitations and lower reactivity of solid reactants.^[^ ^43^, ^44^, ^45^, ^46^ ^]^ The involvement of lattice O* in catalytic soot combustion over K*_x_* WO_3_ was demonstrated using the temperature‐programmed oxidation (TPO) technique in an ^18^O_2_ isotopic atmosphere.^[^ ^47^ ^]^ As shown in **Figure** **6**a,b, the main product during the initial stage was C^16^O_2_, whereas the concentration of C^16^O^18^O clearly increased and became the dominant species as the reaction progressed, confirming that soot oxidation followed the MvK mechanism, known as lattice oxygen actively participates in reactions (Figure 6b inset), in accordance with previous research.^[^ ^48^ ^]^ Then, soot combustion activities were measured by TPO reactions, and the results are shown in Figure 6c. The K_0.37_WO_3_ catalyst shows the highest catalytic activity with *T*_50_ = 504 °C, defined as the temperature for 50% soot conversion, achieving a significant reduction (60 °C) compared to HWO. In the context of TPO reactions, the activity enhancement exhibited by K_0.37_WO_3_ (*T*_50_ = 504 °C) appeared to be less pronounced in comparison to the K_0.32_WO_3_ (*T*_50_ = 517 °C) and K_0.29_WO_3_ (*T*_50_ = 529 °C) samples, attributing to the reduced specific surface area of K_0.37_WO_3_ (7.2 m^2^·g^−1^), as compared to those of K_0.32_WO_3_ (11.1 m^2^·g^−1^) and K_0.29_WO_3_ (11.4 m^2^·g^−1^) (Figure S25, Supporting Information). A performance comparison between our work and recently reported confined potassium catalysts is presented in Table S4 (Supporting Information).

To eliminate the effect of the K content and the specific surface area, the reaction rates were measured in the kinetic range (at 390 °C), and a relationship between the reaction rate per unit area and K content was established. As shown in Figure 6d, the reaction rate exhibited a gradual increase from K_0.29_WO_3_ to K_0.32_WO_3_ when the K content was below 5.26 wt.% (i.e., x<0.33) due to the activation of O_2_ via V_o_ sites generated by K^+^ insertion. As a typical anion defect, V_o_ can directly adsorb molecular O_2_ to form surface O^*^,^[^ ^49^, ^50^ ^]^ which was validated by the strong linear correlation between V_o_ concentration and the intensity of O^*^ amount (O_3_^−^ signal) in quantitative EPR analysis (Figure S26, Supporting Information). Significantly, a substantial improvement in the reaction rate of K_0.37_WO_3_ was observed, suggesting that the K*^δ^* ^+^‐driven activation of the lattice oxygen played a dominant role besides the above conventional V_o_‐derived effects.

More deeply, the intrinsic activity, i.e., turnover frequency (TOF) for soot combustion, was calculated according to the amount of available surface active oxygen (O^*^) and normalized reaction rates by surface areas during the isothermal anaerobic titration of the soot oxidation process.^[^ ^51^ ^]^ As shown in Figure 6e and Figure S27 (Supporting Information), K_0.37_WO_3_ exhibits the largest amount of O^*^ among the three K*_x_* WO_3_ catalysts, providing nearly twice the amount of O^*^ compared with those provided by K_0.32_WO_3_ and K_0.29_WO_3_. Furthermore, the TOF value of K_0.37_WO_3_ (2.47 s^−1^) was much greater than those of K_0.32_WO_3_ (1.96 s^−1^) and K_0.29_WO_3_ (1.83 s^−1^), with the latter two values being quite similar. These results confirm that the similar reaction mechanism between K_0.32_WO_3_ and K_0.29_WO_3_ is based on V_o_‐derived O_2_ activation,^[^ ^48^ ^]^ while the greater amount of higher activity O^*^ in K_0.37_WO_3_ originates from the K*^δ^* ^+^‐driven activation of lattice oxygen. While it is well established that alkali ions enhance the activation of metal‐oxygen bonds via direct electron donation, exerting this specific electron‐promoting effect of alkali ions across a wide range of heterogeneous catalytic systems remains a challenge for future studies.

Cycling stability tests of soot combustion were also evaluated over K*_x_* WO_3_ catalysts subjected to four consecutive thermal cycles, during which the temperature was ramped from 25 to 700 °C. No appreciable degradation in catalytic activity (Figure 6f) and their hexagonal structure (Figure S28 and Note S4, Supporting Information) was observed, indicating excellent stability under the investigated conditions. In contrast, the HWO sample underwent phase transformation only after one soot combustion cycle (Figure S29, Supporting Information), confirming the accumulation of K ions in tunnels stabilizing the WO_3_ skeleton structure. In addition, the hexagonal tunnels of WO_3_ can effectively trap K⁺ ions to prevent their loss, a major deactivation factor observed in surface alkali‐promoted catalysts.^[^ ^52^ ^]^ As confirmed by DFT calculations, the extraction of K ions from the tunnels is a relatively challenging process, exhibiting a higher energy barrier of 0.40 eV (Figure S1, Supporting Information) in comparison to intra‐tunnel K ion migration (0.20 eV) (Figure S30, Supporting Information). Consequently, K ions preferentially migrate along the structural channels rather than escaping the WO_3_ framework, thereby preserving catalyst stability.

The K ion mobility within the tunnels and the deriving force were studied by K 2p near‐ambient pressure (NAP) XPS spectra. As shown in Figure 6g, a clear decrease in the peak intensity as the temperature increases from 25 to 400 °C under reductive CO was observed, suggesting that the consumption of lattice oxygen (formation of V_o_) drives the K ions diffusing outward from the monitored area. However, when the atmosphere switches from CO to oxidative O_2_, the K ion peak intensity rebounds, suggesting that the K^+^ ions move back to their original positions. These results indicate that the migration of K ions within tunnels is a lattice oxygen/V_o_‐driven process (Figure 6h upper). Consequently, the presence of a significant number of V_o_ derived from K ion insertion enhances localized enrichment of K ions in the tunnels that, in turn, facilitates the formation of K^δ+^ species (Figure 6h bottom). Besides, this result first provides direct evidence of the mobility of confined K ions, previously described as quasi‐free K cations in previous work,^[^ ^24^, ^44^ ^]^ which are critical to the triphase soot combustion reaction.

## 3 Conclusion

We constructed single K ion chains confined in hexagonal WO_3_ tunnels via a high‐temperature‐driven ion diffusion‐coordination strategy, aiming to modulate the electronic state of K ions and thus to identify the mechanistic pathway of K ions as electron donors. The unique structure achieved K ion contents that exceed the theoretical limit (*x* > 0.33 in K_*x*_WO_3_) by simultaneous occupation of weak‐stability K_6c_ sites in addition to high‐stability K_12c_ sites, yielding high‐electron‐density K*^δ^* ^+^ ions (0 < δ < 1) in K_0.37_WO_3_, which enables direct electron donation to [WO_6_] motif antibonding orbitals, thereby triggering lattice oxygen activation. However, when the K content is below the theoretical (*x *< 0.33), as in K_0.29_WO_3_ and K_0.32_WO_3_, only K^+^ ions occur that exhibit classical electron effect mediated by V_o_, leading to conventional O_2_ activation pathways. This work provides novel insights into the electron donation mechanisms of alkali ions in catalytic systems. As a proof of concept for heterogeneous catalysis, the K_0.37_WO_3_ catalyst demonstrated superior promotion of the challenging oxidation reaction of soot combustion using O_2_, a key process in automotive catalysis. The present findings may inspire research on other important reactions containing alkali ions as electron‐donor promoters.

## Conflict of Interest

The authors declare no conflict of interest.

## Supporting information



Supporting Information

## Data Availability

Materials and methods, synthetic procedures, and the additional structural characterizations and DFT simulation results can be found in the Supplementary Data.

## References

[1] J. Guo , P. Chen , Acc. Chem. Res. 2021, 54, 2434. 33913703 10.1021/acs.accounts.1c00076

[2] F. Chang , I. Tezsevin , J. W. de Rijk , J. D. Meeldijk , J. P. Hofmann , S. Er , P. Ngene , P. E. de Jongh , Nat. Catal. 2022, 5, 222.

[3] R. Wang , Y. Chen , X. Shang , B. Liang , X. Zhang , H. Zhuo , H. Duan , X. Li , X. Yang , X. Su , Y. Huang , T. Zhang , ACS Catal. 2024, 14, 11121.

[4] M. Wang , P. Wang , G. Zhang , Z. Cheng , M. Zhang , Y. Liu , R. Li , J. Zhu , J. Wang , K. Bian , Y. Liu , F. Ding , T. P. Senftle , X. Nie , Q. Fu , C. Song , X. Guo , Sci. Adv. 2023, 9, adg0167. 10.1126/sciadv.adg0167 PMC10275596 37327337

[5] Q. Yang , V. A. Kondratenko , S. A. Petrov , D. E. Doronkin , E. Saraci , H. Lund , A. Arinchtein , R. Kraehnert , A. S. Skrypnik , A. A. Matvienko , E. V. Kondratenko , Angew. Chem., Int. Ed. 2022, 61, 202116517. 10.1002/anie.202116517 PMC9314630 35244964

[6] C. F. Huo , B. S. Wu , P. Gao , Y. Yang , Y. W. Li , H. Jiao , Angew. Chem., Int. Ed. 2011, 50, 7403. 10.1002/anie.201007484 21714044

[7] M. Yang , S. Li , Y. Wang , J. A. Herron , Y. Xu , L. F. Allard , S. Lee , J. Huang , M. Mavrikakis , M. Flytzani‐Stephanopoulos , Science 2014, 346, 1498. 25431492 10.1126/science.1260526

[8] J. W. Doebereiner , Ann. Phys. 1845, 140, 94.

[9] W. D. Mross , Catal. Rev. 1983, 25, 591.

[10] Z. Hao , Z. Shen , Y. Li , H. Wang , L. Zheng , R. Wang , G. Liu , S. Zhan , Angew. Chem., Int. Ed. 2019, 58, 6351. 10.1002/anie.201901771 30882987

[11] A. Lucas‐Consuegra , Catal. Surv. Asia 2014, 19, 25.

[12] S. R. Bare , D. R. Strongin , G. A. Somorjai , J. Phys. Chem. 1986, 90, 4726.

[13] J.‐H. Kim , T.‐Y. Dai , M. Yang , J.‐M. Seo , J. S. Lee , D. H. Kweon , X.‐Y. Lang , K. Ihm , T. J. Shin , G.‐F Han , Q. Jiang , J.‐B. Baek , Nat. Commun. 2023, 14, 2319. 37087491 10.1038/s41467-023-38050-2 PMC10122650

[14] J. Xie , P. P. Paalanen , T. W. van Deelen , B. M. Weckhuysen , M. J. Louwerse , K. P. de Jong , Nat. Commun. 2019, 10, 167. 30635560 10.1038/s41467-018-08019-7 PMC6329823

[15] R. Qin , L. Zhou , P. Liu , Y. Gong , K. Liu , C. Xu , Y. Zhao , L. Gu , G. Fu , N. Zheng , Nat. Catal. 2020, 3, 703.

[16] Q. Zhang , S. Gao , J. Yu , Chem. Rev. 2023, 123, 6039. 36049046 10.1021/acs.chemrev.2c00315

[17] Y. Chen , D. Tang , Z. Huang , X. Liu , J. Chen , T. Sekiguchi , W. Qu , J. Chen , D. Xu , Y. Bando , X. Hu , X. Wang , D. Golberg , X. Tang , Nat. Commun. 2021, 12, 1191. 33608540 10.1038/s41467-021-21462-3 PMC7895918

[18] Z. Huang , H. Li , J. Gao , X. Gu , L. Zheng , P. Hu , Y. Xin , J. Chen , Y. Chen , Z. Zhang , J. Chen , X. Tang , Environ. Sci. Technol. 2015, 49, 14460. 26587749 10.1021/acs.est.5b03972

[19] V. P. Santos , O. S. G. P. Soares , J. J. W. Bakker , M. F. R. Pereira , J. J. M. Órfão , J. Gascon , F. Kapteijn , J. L. Figueiredo , J. Catal. 2012, 293, 165.

[20] Y. Chen , G. Tian , M. Zhou , Z. Huang , C. Lu , P. Hu , J. Gao , Z. Zhang , X. Tang , Environ. Sci. Technol. 2016, 50, 5825. 27128185 10.1021/acs.est.5b06109

[21] Z. Zheng , B. Yan , J. Zhang , Y. You , C. T. Lim , Z. Shen , T. Yu , Adv. Mater. 2008, 20, 352.

[22] L. Li , F. Jiang , F. Tu , S. Jia , Y. Gao , J. Wang , Adv. Sci. 2017, 4, 1600537. 10.1002/advs.201600537 PMC5604398 28932660

[23] C. Guo , S. Yin , L. Huang , T. Sato , ACS Appl. Mater. Interfaces 2011, 3, 2794. 21675747 10.1021/am200631e

[24] T. Liu , Q. Li , Y. Xin , Z. Zhang , X. Tang , L. Zheng , P.‐X. Gao , Appl. Catal. B‐Environ. 2018, 232, 108.

[25] L. Pauling General Chemistry, Dover Publications, Garden City, NY, USA 1988.

[26] D. Jose , A. Datta , J. Phys. Chem. C 2012, 116, 24639.

[27] K. Pokhodnya , C. Olson , X. Dai , D. L. Schulz , P. Boudjouk , A. P. Sergeeva , A. I. Boldyrev , J. Chem. Phys. 2011, 134, 014105. 21218995 10.1063/1.3516179

[28] A. S. Ivanov , E. Miller , A. I. Boldyrev , Y. Kameoka , T. Sato , K. Tanaka , J. Phys. Chem. C 2015, 119, 12008.

[29] I. B. Bersuker , Chem. Rev. 2021, 121, 1463. 33353296 10.1021/acs.chemrev.0c00718

[30] S. Yamazoe , Y. Hitomi , T. Shishido , T. Tanaka , J. Phys. Chem. C 2008, 112, 6869.

[31] U. Jayarathne , P. Chandrasekaran , A. F. Greene , J. T. Mague , S. DeBeer , K. M. Lancaster , S. Sproules , J. P. Donahue , Inorg. Chem. 2014, 53, 8230. 25068843 10.1021/ic500256a PMC4139175

[32] C. H. Jia , X. P. Xiang , J. Zhang , Z. Y. He , Z. H. Gong , H. J. Chen , N. Zhang , X. W. Wang , S. J. Zhao , Y. Chen , Adv. Funct. Mater. 2023, 33, 2301981

[33] G. L. Stamokostas , G. A. Fiete , Phys. Rev. B. 2018, 97, 085150.

[34] J. Purans , A. Kuzmin , P. Parent , Phys. B 1999, 259, 1157.

[35] F. Frati , M. Hunault , F. M. F. de Groot , Chem. Rev. 2020, 120, 4056. 32275144 10.1021/acs.chemrev.9b00439 PMC7227067

[36] R. Brunetti , C. Jacoboni , M. Rudan , J. Appl. Phys. 2024, 136, 085701.

[37] N. N. Greenwood , A. Earnshaw , Chemistry of the Elements, 2nd ed., Butterworth‐Heinemann, Oxford, UK 1997.

[38] J. Purans , A. Kuzmin , P. Parent , C. Laffon , Electrochim. Acta 2001, 46, 1973.

[39] S. Yoshio , M. Okada , K. Adachi , J. Appl. Phys. 2018, 124, 063109.

[40] M. Okada , K. Ono , S. Yoshio , H. Fukuyama , K. Adachi , J. Am. Ceram. Soc. 2019, 102, 5386.

[41] Y. Hinuma , T. Toyao , T. Kamachi , Z. Maeno , S. Takakusagi , S. Furukawa , I. Takigawa , K. Shimizu , J. Phys. Chem. C 2018, 122, 29435.

[42] D. Zheng , K. Liu , Z. Zhang , Q. Fu , M. Bian , X. Han , X. Shen , X. Chen , H. Xie , X. Wang , X. Yang , Y. Zhang , S. Song , Nat. Commun. 2024, 15, 6688. 39107273 10.1038/s41467-024-51034-0 PMC11303551

[43] X. Mei , X. Zhu , Y. Zhang , Z. Zhang , Z. Zhong , Y. Xin , J. Zhang , Nat. Catal. 2021, 4, 1002.

[44] P. Legutko , P. Stelmachowski , X. Yu , Z. Zhao , Z. Sojka , A. Kotarba , ACS Catal. 2023, 13, 3395.

[45] Y. Li , T. Qin , Y. Wei , J. Xiong , P. Zhang , K. Lai , H. Chi , X. Liu , L. Chen , X. Yu , Z. Zhao , L. Li , J. Liu , Nat. Commun. 2023, 14, 7149. 37932256 10.1038/s41467-023-42935-7 PMC10628289

[46] R. Kimura , J. Wakabayashi , S. P. Elangovan , M. Ogura , T. Okubo , J. Am. Chem. Soc. 2008, 130, 12844. 18774818 10.1021/ja802102q

[47] B. A. A. L. van Setten , M. Makkee , J. A. Moulijn , Catal. Rev. 2001, 43, 489.

[48] C. Wang , H. Y. Yuan , G. Z. Lu , H. F. Wang , Appl. Catal. B‐Environ. 2021, 281, 119468.

[49] J. Yang , S. Hu , Y. Fang , S. Hoang , L. Li , W. Yang , Z. Liang , J. Wu , J. Hu , W. Xiao , C. Pan , Z. Luo , J. Ding , L. Zhang , Y. Guo , ACS Catal. 2019, 9, 9751.

[50] Y. Zheng , K. Fu , Z. Yu , Y. Su , R. Han , Q. Liu , J. Mater. Chem. A 2022, 10, 14171.

[51] Z. Zhang , D. Han , S. Wei , Y. Zhang , J. Catal. 2010, 276, 16.

[52] Q. Li , X. Wang , H. Chen , Y. Xin , G. Tian , C. Lu , Z. Zhang , L. Zheng , L. Zheng , Catal. Today 2016, 264, 171.

