# Supplementary material for: Unraveling Alkali Ion Electron Donation for Enhancing Heterogeneous Catalytic Oxidation
Source: Adv Sci (Weinh). 2025 Oct 8;13(2):e14470. doi: 10.1002/advs.202514470 (PMC12786274; doi:10.1002/advs.202514470)
Supplement: Supplementary file 1 — Supporting Information [file ADVS-13-e14470-s001.docx]

Supporting Information

Unraveling Alkali Ion Electron Donation for Enhancing Heterogeneous Catalytic Oxidation

Jin Wang^a^, Zhenghui Zhang^a^, Xianqiu Song^a^, Rong Wang^a^, Ying Xin^a,^ *, Yexin Zhang^b,c^ and Zhaoliang Zhang ^a,^ *

**Supplementary Methods**

**Synthesis of catalysts**

Hexagonal WO_3_ nanorods were synthesized by using a facile hydrothermal method. 0.7 mmol ammonium metatungstate ((NH_4_)_6_W_12_O_39_·*x*H_2_O), 63.0 mmol ammonium sulfate ((NH_4_)_2_SO_4_) and 23.3 mmol oxalic acid (H_2_C_2_O_4_·2H_2_O) was dissolved in ultrapure water (80 mL). The solution was transferred to a 100 mL Teflon-lined autoclave and heated at 180 ^o^C for 12 h in homogeneous reactor with 20 *r*·min^-1^. The product obtained was washed with ultrapure water several times until the pH value to ~7 in order to remove the hetero-ions, then dried at 105 °C for 12 h, and then calcined in air at 350 °C for 4 h. The synthesized sample is denoted as HWO. The impregnation method was adopted to obtain the K-HWO samples. Typically, 2g HWO powder was dispersed in KNO_3_ aqueous solution (3.1mmol/20mL) and sonicated for 10 min. The obtained slurry was heated to 80 ^o^C, and then evaporated with continuous stirring. The product was transfer to drying oven and dried at 105^o^C for 12h, and then calcined in air at 350 °C for 12 h, followed by calcination at 500 °C for 2 h. Finally, the product was washed with ultrapure water at room temperature for 24 h to remove the surface K^+^, and then dried at 105^o^C for 12h. The synthesized sample is denoted as K_0.37_WO_3_, *x* is values determined by Inductively coupled plasma optical emission spectroscopy (ICP-OES). By decrease the ratio of KNO_3_ to HWO, the control catalysts of K_0.29_WO_3_ and K_0.32_WO_3_ were also obtained. The samples after the cycling test are respectively designated as K_0.29_WO_3_-A and K_0.32_WO_3_-A and K_0.37_WO_3_-A, respectively.

**Characterizations**

Inductively coupled plasma optical emission spectroscopy (ICP-OES) was used as an elemental analysis technology to detect the content of K on an OPTIMA2100DV instrument from PerkinElmer.

X-ray powder diffraction (XRD) patterns were measured on a D8 FOCUS powder X-ray diffraction instrument (Bruker AXS, Germany) using 40 kV as tube voltage and 40 mA as tube current. To obtain detailed information about the atomic structure of crystalline materials, Synchrotron X-ray diffraction (SXRD) patterns were recorded utilizing high brilliance synchrotron sources at beamline 4B9A of Beijing Synchrotron Radiation Facility (BSRF). Rietveld analysis (structure profile refinements) of the diffraction data was performed with the FullProf software package.

Field-emission scanning electron microscopy (FESEM) equipped with energy dispersive spectroscopy (EDS) was performed on a Hitachi SU-70 microscope.

High-angle annular dark-field (HAADF) images and elemental maps with atomic resolution were obtained using JEM-ARM300F, a 300 kV atomic resolution transmission electron microscope featuring JEOL's own aberration correctors, equipped with energy dispersive spectroscopy (EDS).

X-ray absorption measurements of the O *K*-edge and W *L_3_*-edge were performed in transmission mode at beamline 4B9B and 1W1B of Beijing Synchrotron Radiation Facility (BSRF), respectively.

Low-temperature electron paramagnetic resonance (EPR) spectra were acquired on a Bruker A300 EPR spectrometer under high vacuum. The samples were pretreated under vacuum condition at 400 °C to remove adsorbed surface oxygen species. To detect the evolution of surface-adsorbed oxygen species, the EPR spectra were collected after exposure to air at 77 K.

Raman spectra were obtained using a LabRAM HR Evolution instrument (Horiba) with the 532 nm laser line. Pretreatment of samples was carried out in the flow of N_2_ for 60 min at 200 ^o^C.

X-ray photoelectron spectroscopy (XPS) data were obtained on a Thermo Scientific ESCALAB 250XI electron spectrometer, using monochromatic Al K_α_ as the exciting radiation.

The mobility of K species in catalysts was characterized in a SPECS near-ambient pressure X-ray photoelectron spectroscopy (NAP-XPS) system under different conditions. The atmosphere includes vacuum condition, O_2_ (99.999%, 1.0 mL·min^-1^) CO (99.9%, 1.0 mL·min^-1^). The binding energy (BE) values were calibrated with the signal of contaminated carbon C1s at 284.8 eV. The spectra were deconvoluted using XPSPEAK software. Surface area and pore size distribution were determined by N_2_ adsorption/desorption at 77 K using

Brunauer-Emmett-Teller (BET) method with a Micromeritics ASAP 2020 instrument after out-gassing for 5 h at 300 ^o^C prior to analysis.

Soot temperature programmed reduction (soot-TPR) experiments were conducted using a TP-5076 multiple adsorption instrument. Typically, 55 mg mixture (5 mg soot and 50 mg powdered catalyst) was placed in a quartz reactor and pretreated in Ar flow for 60 min at 120 ^o^C to remove any physically adsorbed impurities. Then, soot-TPR was carried out from 50 to 700 ^o^C with a heating rate of 5 ^o^C·min^-1^ in the flow of He (100 mL·min^-1^). A thermal conductivity detector (TCD) was employed to monitor the CO and CO_2_ formation.

Temperature programmed reduction with H_2_ (H_2_-TPR) experiments were performed in a quartz reactor with a thermal conductivity detector (TCD). Typically, a 50 mg sample was pretreated in situ for 30 min. at 120 °C in a flow of Ar (30 ml·min^-1^) and cooled to room temperature. TPR was conducted at 10 °C/min up to 800 °C in a 30 mL·min^-1^ flow of 5 vol.% H_2_/N_2_.

**Catalytic activity**

Temperature-programmed oxidation (TPO) reactions were conducted in a fixed bed micro-reactor using Printex-U from Degussa as the model soot. In a typical procedure, grind the mixture of soot and catalyst (weight ratio of catalyst/soot = 9:1) in an agate mortar for 30 min to obtain a homogeneous mixture. 50 mg sample of the soot-catalyst mixture was pretreated in a flow of He (100 mL·min^-1^) at 200 ^o^C for 30 min to remove adsorbed species. Then, a gas flow with 5 vol.% O_2_ in He was introduced with a heating rate of 5 ^o^C·min^-1^ from room temperature to 700 ^o^C. The composition of the gases was monitored online by gas chromatography.

Isotopic isothermal reaction was performed by switching the flowing gas from 1% ^16^O_2_ to 1% ^18^O_2_ diluted in Ar at 200 ^o^C followed by TPO reactions in a fixed bed micro-reactor. 50 mg of a mixture of the soot and catalyst in tight contact mode was employed. The effluent gas from the reactor was continuously monitored by a MS for all of the isotopic molecules of CO_2_ (at m/z = 44, 46 and 48) and CO (at m/z = 28 and 30) from 300 to 700 ^o^C.

The selectivity to CO_2_ (*S*_CO2_) is defined as the percentage CO_2_ in the outlet concentration divided by the sum of the CO_2_ and CO outlet concentrations. To investigate the stability of as-prepared catalysts, the catalytic performance of the catalyst with the four-cycle reaction was also measured.

The intrinsic activity, turnover frequency (TOF), is measured by an isothermal anaerobic titration with soot as a probe molecule, as suggested previously^[1]^. A 50 mg mixture of catalyst and soot (9:1) was diluted with 100 mg silica (below 300 mesh). After pretreatment in a flow of He (100 mL·min^-1^) at 120 °C for 30 min, a gas flow with 5 vol.% O_2_ in He (150 mL·min^-1^) was introduced. The isothermal reaction rates were detected at 390 °C in an approximate kinetic regime due to the soot conversion was stable and low but sufficient for analysis purposes, which followed by anaerobic titration with a switch of atmosphere from O_2_ to He. The transient decay in concentrations was monitored using a quadruple mass spectrometer (MS, OmniStar 200, Balzers). The number of active redox sites available to soot under these reaction conditions can be quantified by integrating the diminishing rate of CO*_x_* formation over time. The reaction rates of catalysts were obtained through isothermal reaction from the slope of the conversion lines with time. The intrinsic activity for soot combustion is obtained via specific rates normalized by BET surface areas.

**Computational details.**

All DFT calculations were performed using the Vienna ab initio simulation package (VASP 5.3.5). The projector augmented wave (PAW) potential and the generalized gradient approximation PBE exchange-correlation functional were used to describe the electron interactions with an energy cut-off of 450 eV. The *k*-point sampling was generated by following the Monkhors-Pack procedure with single k-point (gamma point). All structures were calculated until the differences in total energy and in self-consistent forces were lower than 10^-4^ eV and 0.02 eV·A^-1^, respectively. The diffusion energy barrier of K in the WO_3_ tunnels were calculated by using the climbing-image nudged elastic band method (CI-NEB) at a reduced force threshold of 0.05 eV·A^-1^. These systems were placed in periodic supercells (3×3) with several atomic layers were employed.

To extract chemical bonding information from DFT calculations, the projected crystal orbital Hamilton populations (pCOHP) method was employed using the local orbital basis suite towards electronic-structure reconstruction (LOBSTER) code ^[2,3]^. The electron localization function (ELF) isosurface analysis were performed on the calculated charge density files (CHGCAR) using VESTA software.

**Supplementary Figures**


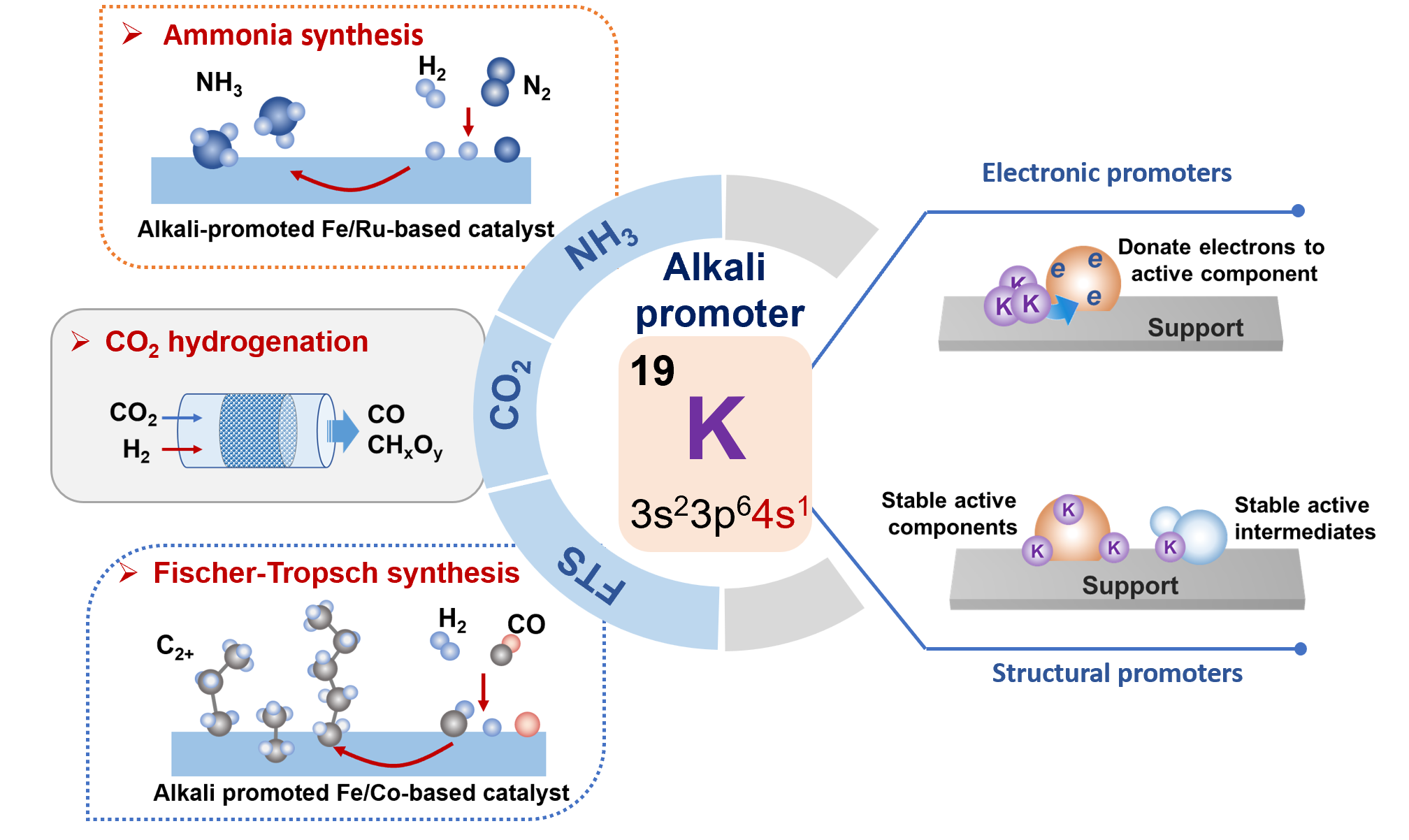


**Illustration S1.** The representative applications and effects of alkali promoter (K as an example).


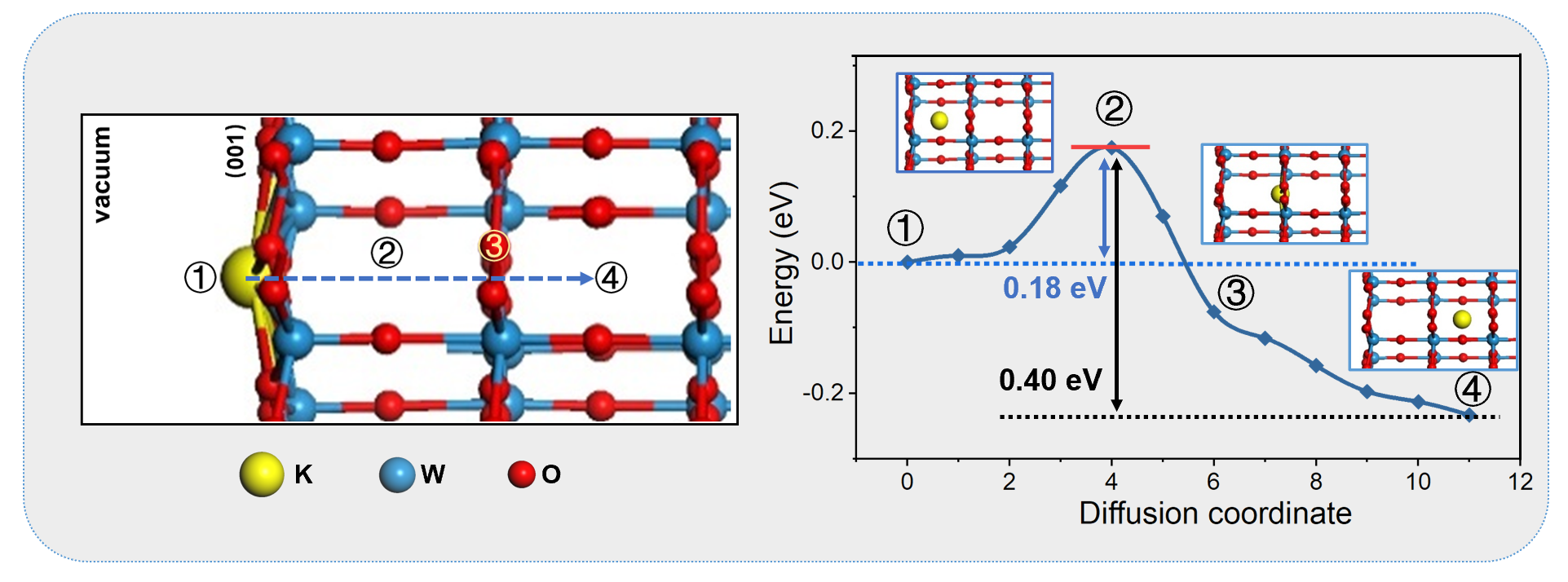


**Figure S1**. The migration trajectories (left) and the corresponding energy profiles (right) of K ion migration from the (001) surface into the hexagonal tunnel. Inset: the seria structures of K ions located at several sites in tunnel.


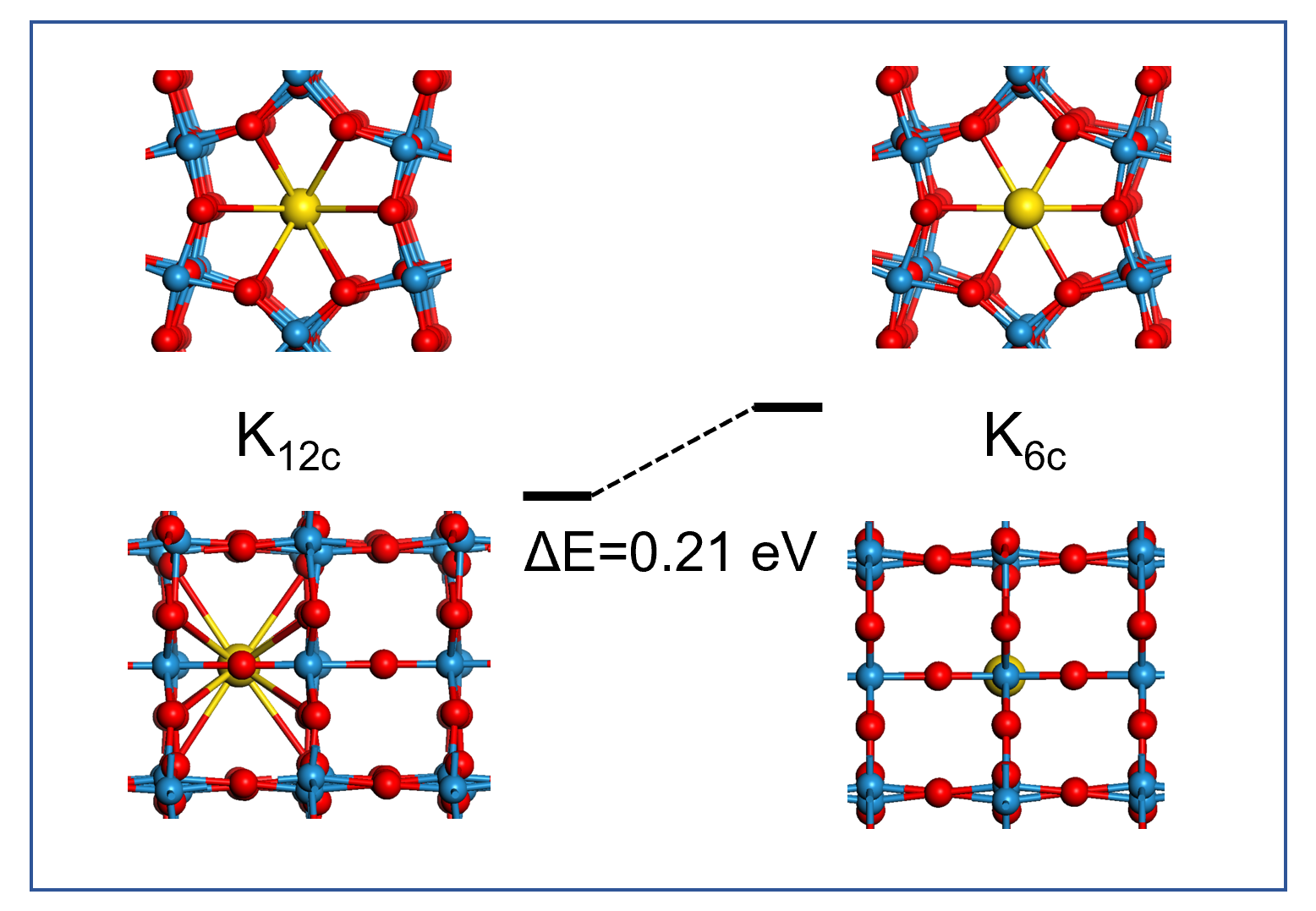


**Figure S2**. Two stable theoretical geometrical configurations of K-ion in the HWO tunnels, and the energy difference of configurations with K ions located on the two sites. K_12c_ and K_6c_ represents the K ions coordinated by twelve and six oxygen atoms, respectively.

**Note S1**: For the available tunnel positions in HWO, K ions preferentially occupy the K_12c_ sites, where each K ion is coordinated by twelve oxygen atoms on the walls of hexagonal-prism tunnels (Figure S1 left). The complete filling of K_12c_ sites would result in the formation of K_0.33_WO_3_^[4-6]^. However, density function theory (DFT) simulations revealed that K ions could be also stabilized by coordinating with six oxygen atoms. The stability of K_6c_ configuration is only slightly lower than that of K_12c_ configuration with an energy difference (Δ*E*) of 0.21 eV (Figure S1 right). Thus, it is possible to exceed the theoretical limit (x>0.33) by creating a few K_6c_ configurations, contributing to a shorter K‒K distance, increased mobility, and then a distinct electronic state.


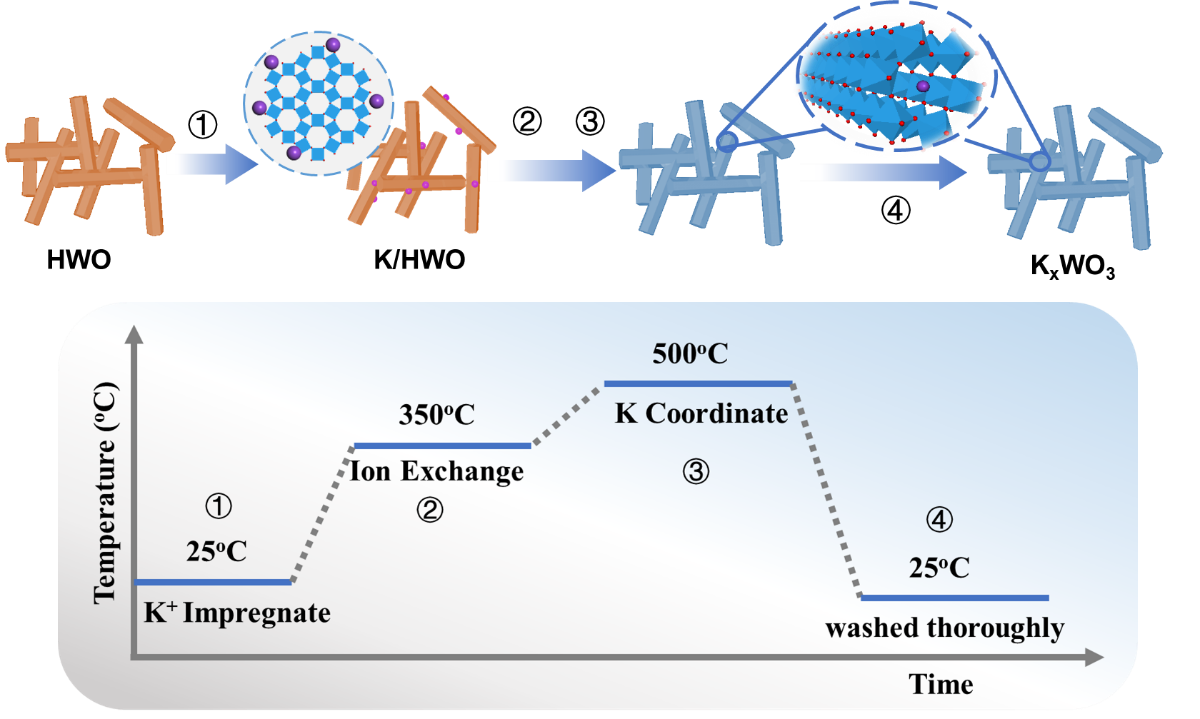


**Figure S3**. Schematic illustration of the preparation of the K*_x_*WO_3_ (*x*=0.29, 0.32 and 0.37) catalysts using KNO_3_ as the K precursor.

**Note S2:** In this methodology, the hexagonal tungsten trioxide (HWO) with pretreatment of KNO_3_ impregnation (K/HWO) serves as the precursor. During Stage 1 (the impregnation process), the slurry of HWO powder dispersed in an aqueous KNO_3_ solution was heated to 80 ℃ under continuous stirring to evaporate the water. Subsequently, the K/HWO precursor was obtained after dried at 105 ℃. This step is conducted at a relatively low temperature to ensure the uniform adsorption of K⁺ ions on the surface of the HWO. Stage 2 is conducted at 350 °C, a temperature specifically selected to facilitate the diffusion and insertion of K^+^ ions into the hexagonal tunnels of HWO. The confined K ions preferentially occupy the K_12c_ sites, where each K ion is coordinated by twelve oxygen atoms. At the same time, it remains sufficiently moderate to prevent the collapse of the metastable HWO crystal structure.

Once a sufficient amount of K^+^ has been incorporated in tunnels during Stage 2, the HWO framework gains enhanced thermal stability. As such, the elevated temperature (500 °C) was employed in Stage 3 to increase the K content in K*_x_*WO_3_ exceeding the theoretical limit (i.e., x > 0.33). So, a higher temperature is necessary to urge the additional K⁺ ions into the tunnels to achieved a high-concentration confined K catalyst (K_0.37_WO_3_).

Finally, the surface potassium ions are removed through a water washing process. The variations among the series of K*_x_*WO_3_ (*x*=0.29, 0.32 and 0.37) catalysts differ only in the amount of impregnated KNO_3_ during the synthesis process.

In various synthetic methods reported previously, the K content has been consistently low. Zheng et al. utilized fresh tungsten foils as both reagents and substrates for the growth of potassium tungsten bronze at 450 °C, following sonication of the tungsten foil in potassium hydroxide (KOH) solution. The resulting compound exhibits a *x* = 0.33 in the K*_x_*W_0.944_O_3_, attaining the maximum K content ^[5]^. Guo et.al employed a hydrothermal synthesis method to obtain one-dimensional potassium tungsten bronze, achieving a *x* = 0.27 in K*_x_*WO_3+_*_x_*_/2_^[7]^. Hou et al. demonstrated that the K^+^ concentration within the tunnels of α-MnO_2_ nanorods could be modulated by varying the KNO_3_ precursor content. However, despite an initial K/Mn atomic ratio of up to 4, the final ratio in the synthesized sample was only 0.08 ^[8]^. These reports highlight the advantage of our method in achieving theoretical limit-breakthrough K content in the tunnels.


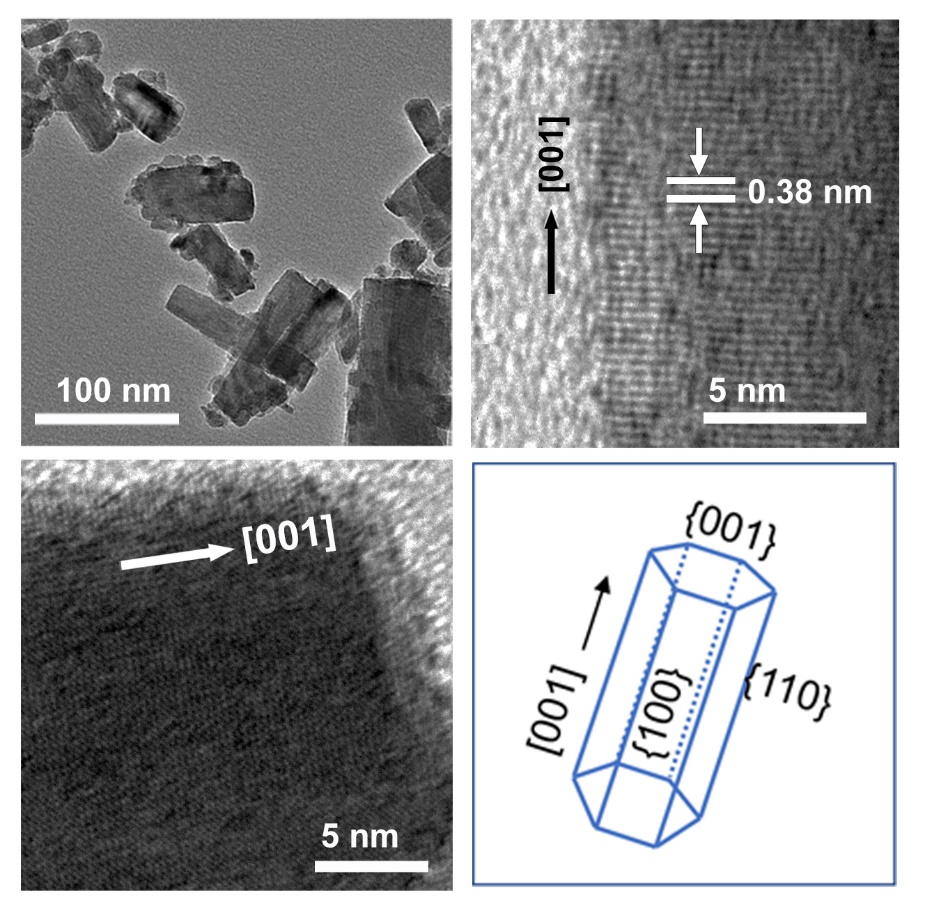


**Figure S4.** TEM and HRTEM images of K_0.37_WO_3_ and the geometrical structural model.


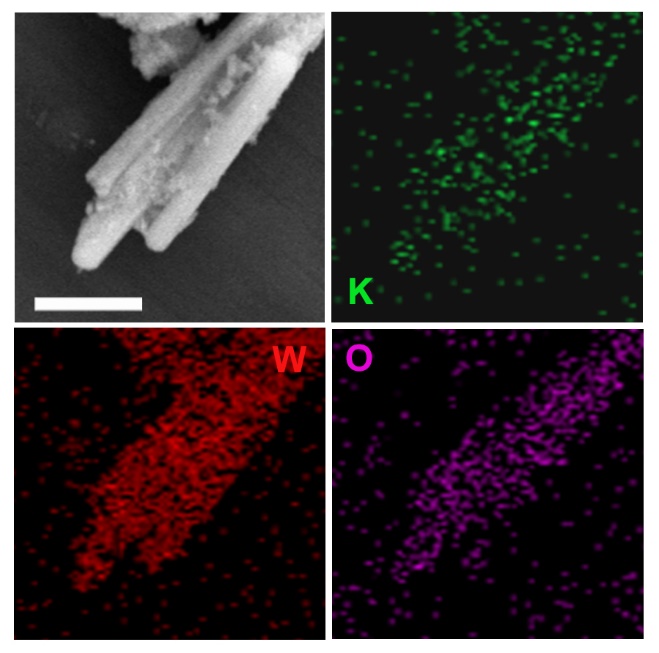


**Figure S5.** SEM image and the element mappings of K, W and O for K_0.37_WO_3_.


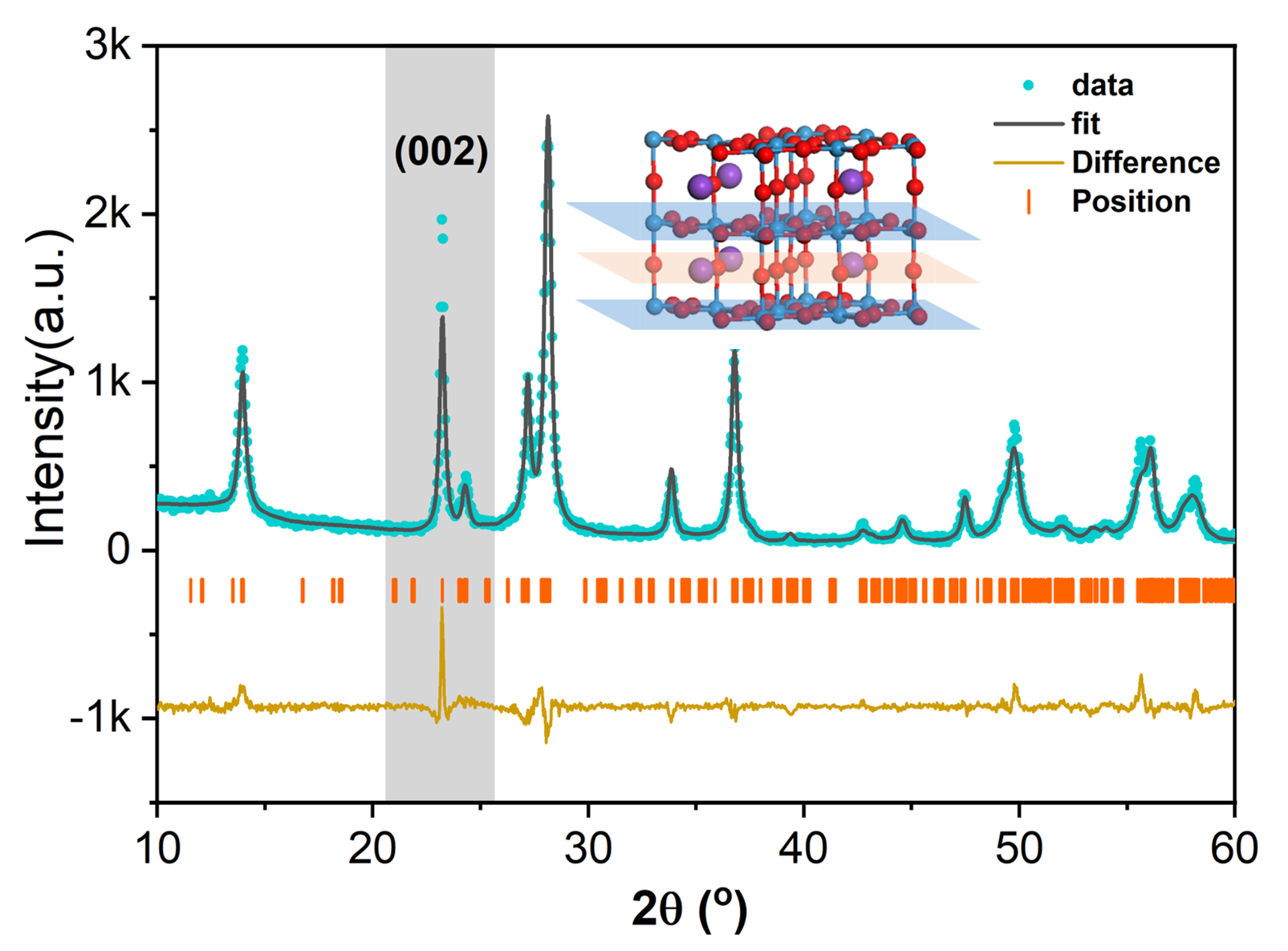


**Figure S6.** Rietveld refinement of K_0.37_WO_3_; the yellow line represents the differential SXRD pattern, and the orange lines indicate the positions of all possible Bragg reflections. The inset shows the (002) plane. The unusually increased (002) peak intensity is attributed to the superposition of the diffraction peaks of the K ion planar plane and the (002) lattice plane of WO_3_ at the same position.


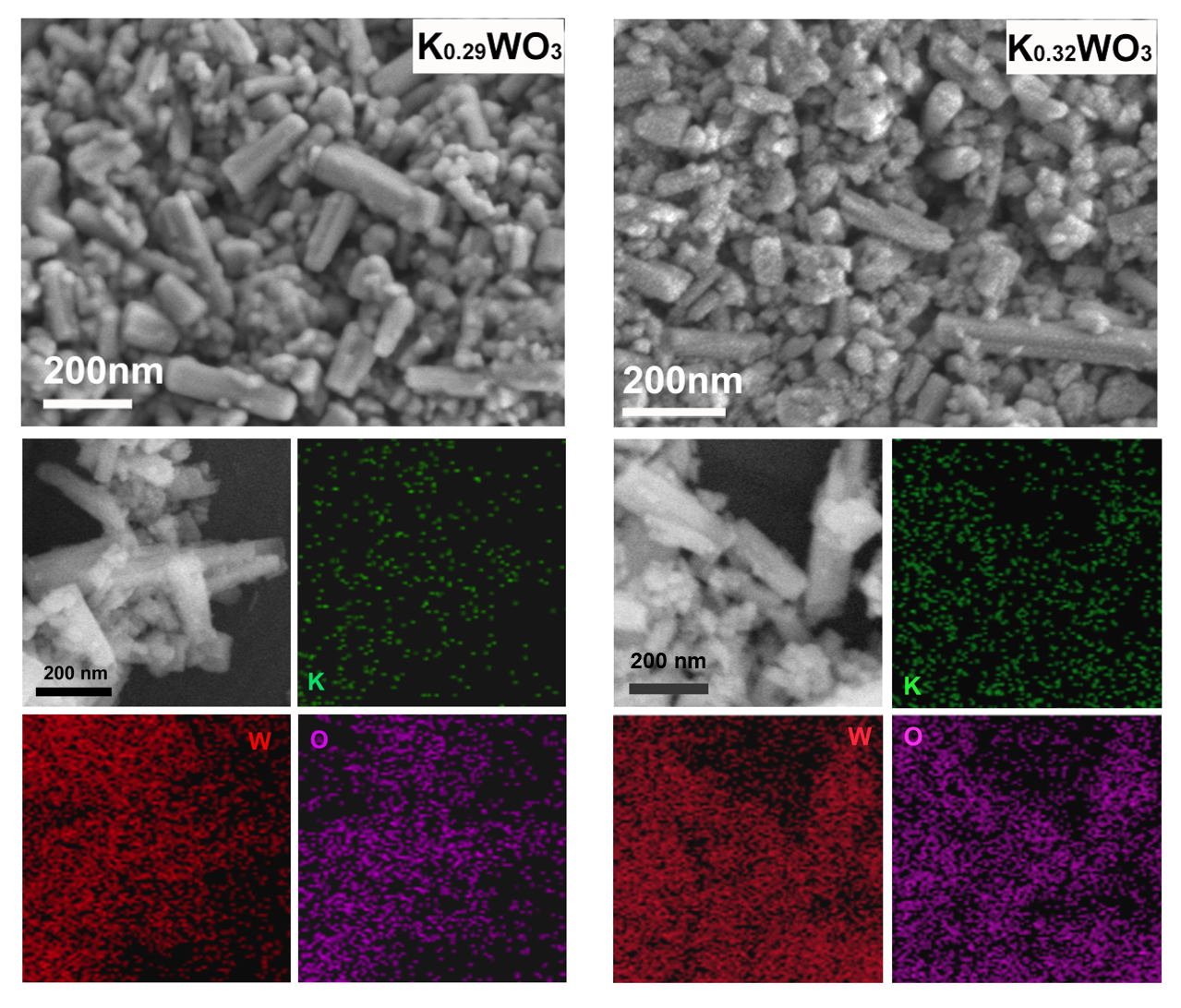


**Figure S7.** SEM images and element mappings of K, W and O for K_0.29_WO_3_ and K_0.32_WO_3_. catalysts.


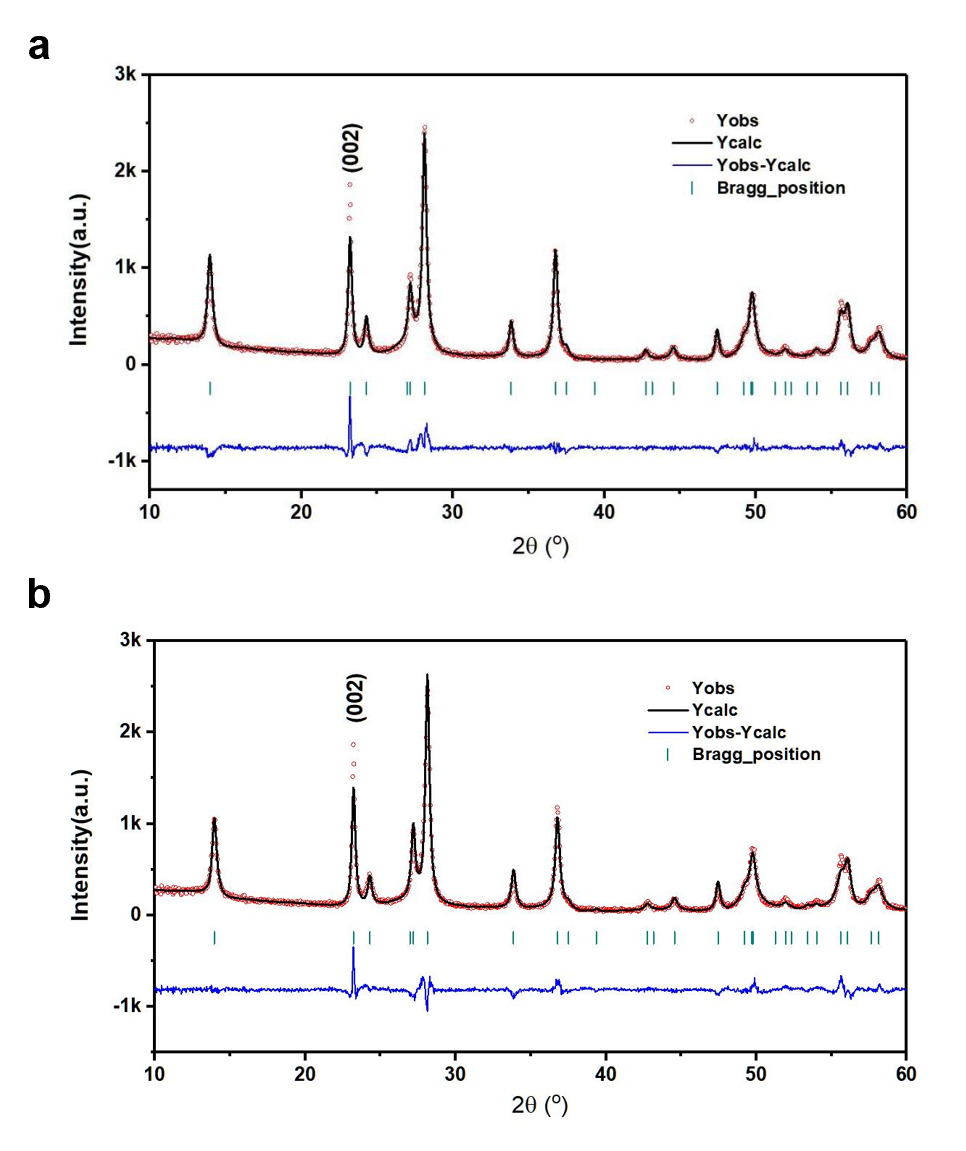


**Figure S8.** SXRD patterns (red dot line) and Rietveld refinements (black line) of (a) K_0.29_WO_3_ and (b) K_0.32_WO_3_. The blue curve is the differential SXRD pattern. The short vertical lines mark the peak positions for all the possible Bragg reflections.


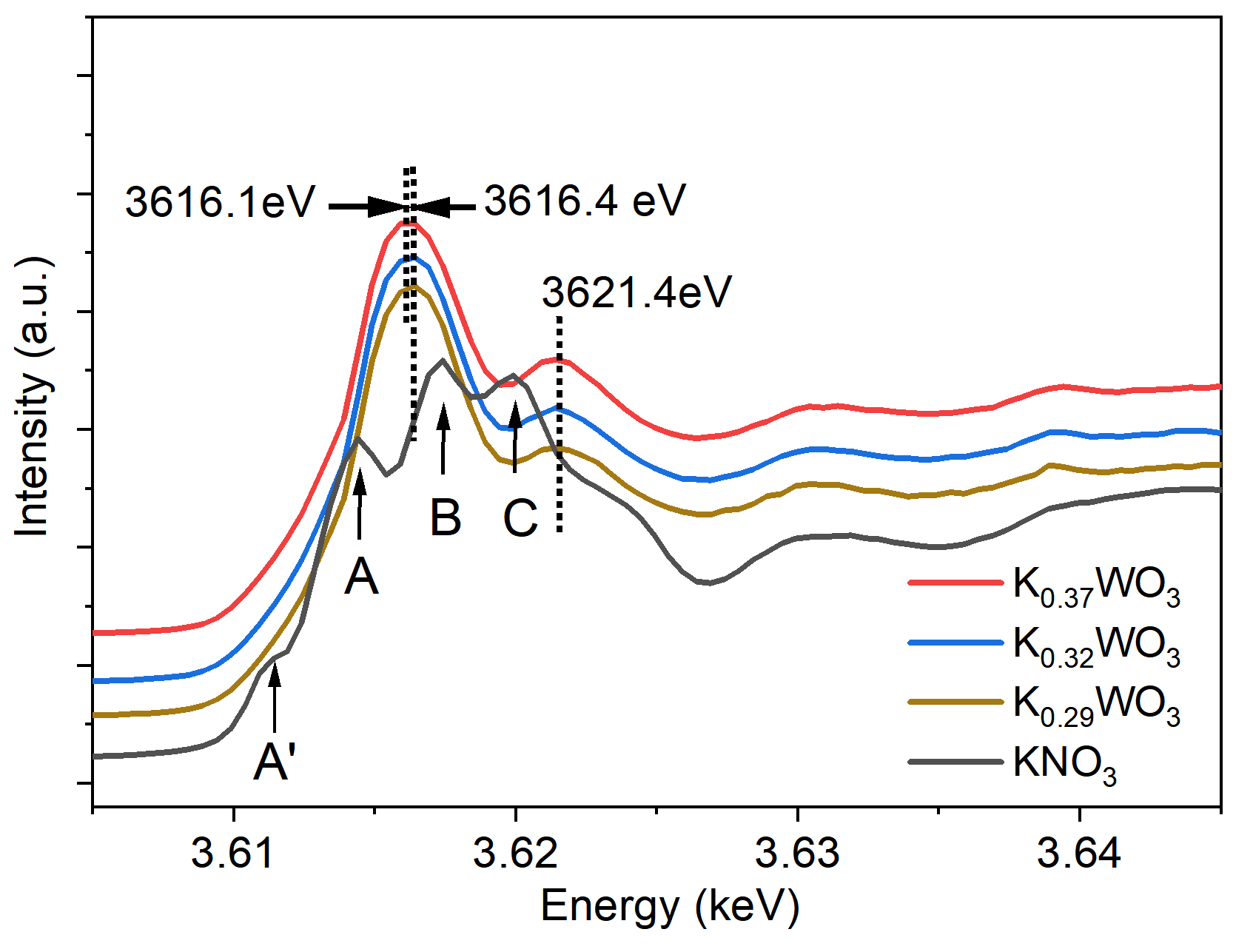


**Figure S9.** K *K*-edge XANES spectra of K*_x_*WO_3_ (*x*=0.29, 0.32 and 0.37) and KNO_3_ precursor solid crystal. The spectrum of KNO_3_ presents multi-peak features with a pre-edge peak at 3611.4 eV (A’), a white line shoulder peak at 3614.4 eV (A), a white line peak at 3614.3 eV (B), and a strong peak after white line at 3619.9.7 eV (C).


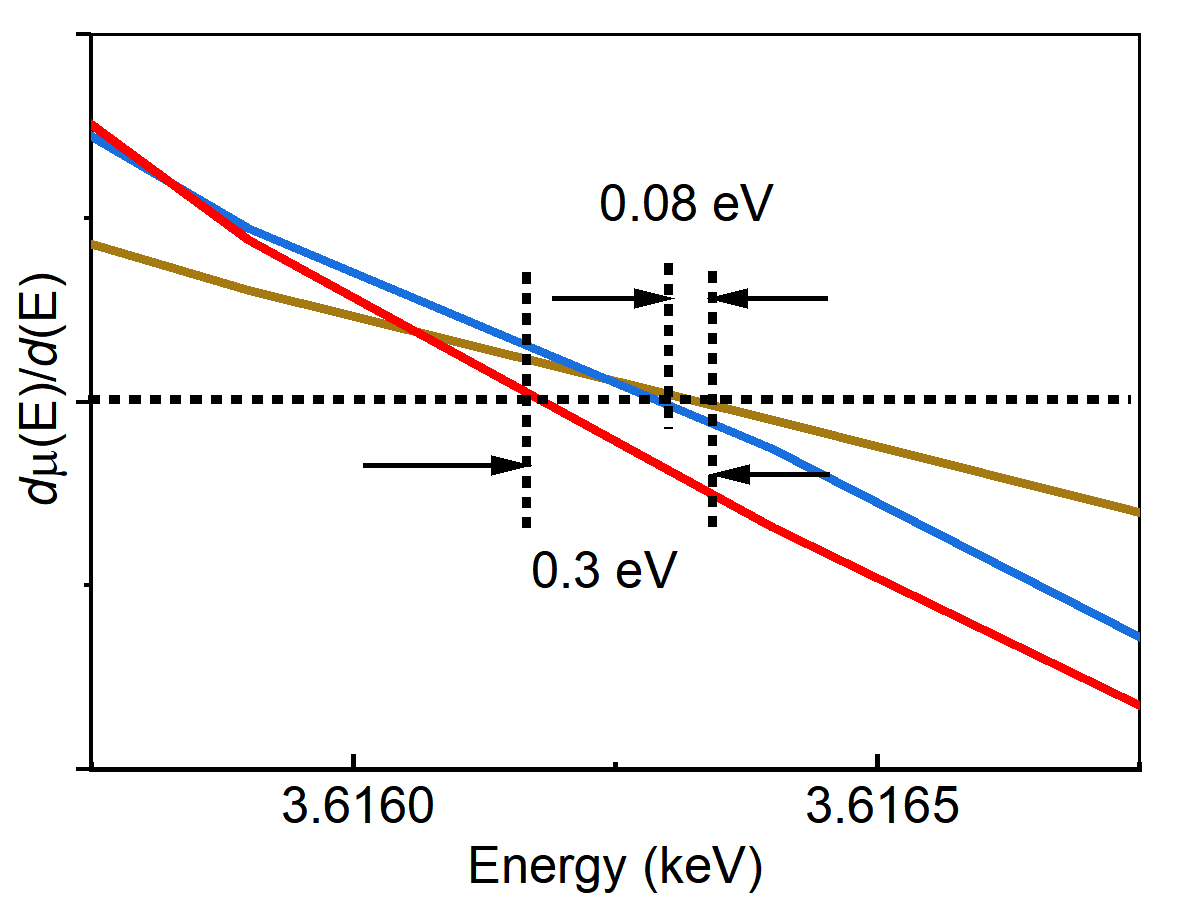


**Figure S10.** The corresponding first derivative spectra of K *K*-edge XANES spectra of K_0.29_WO_3_ (green), K_0.29_WO_3_ (blue) and K_0.29_WO_3_ (red).


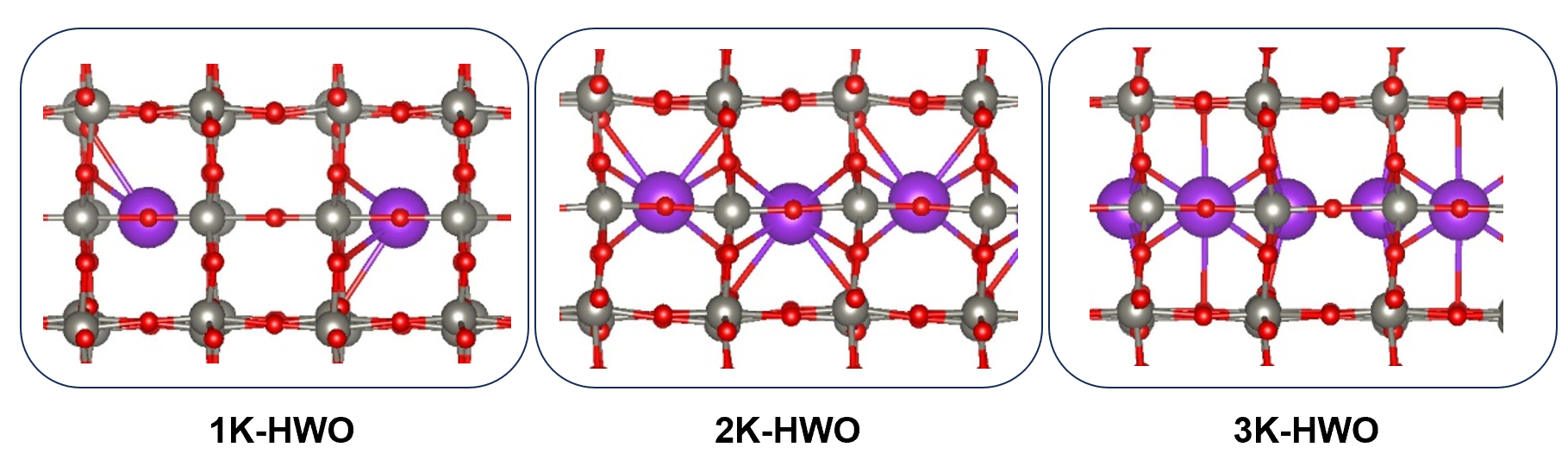


**Figure S11**. The geometric structures of three models, 1K-HWO, 2K-HWO and 3K-HWO, which represent the K_0.29_WO_3_, K_0.32_WO_3_ and K_0.37_WO_3_, respectively. In the 2K-HWO structural model, all K_12c_ sites are fully occupied by potassium atoms. To construct the 1K-HWO model, partial K atoms were selectively removed from the K_12c_ sites followed by structural relaxation. For the 3K-HWO model, additional K atoms were introduced into the hexagonal tunnels of the 2K-HWO framework, which subsequently occupied K_6c_ sites after structural relaxation.


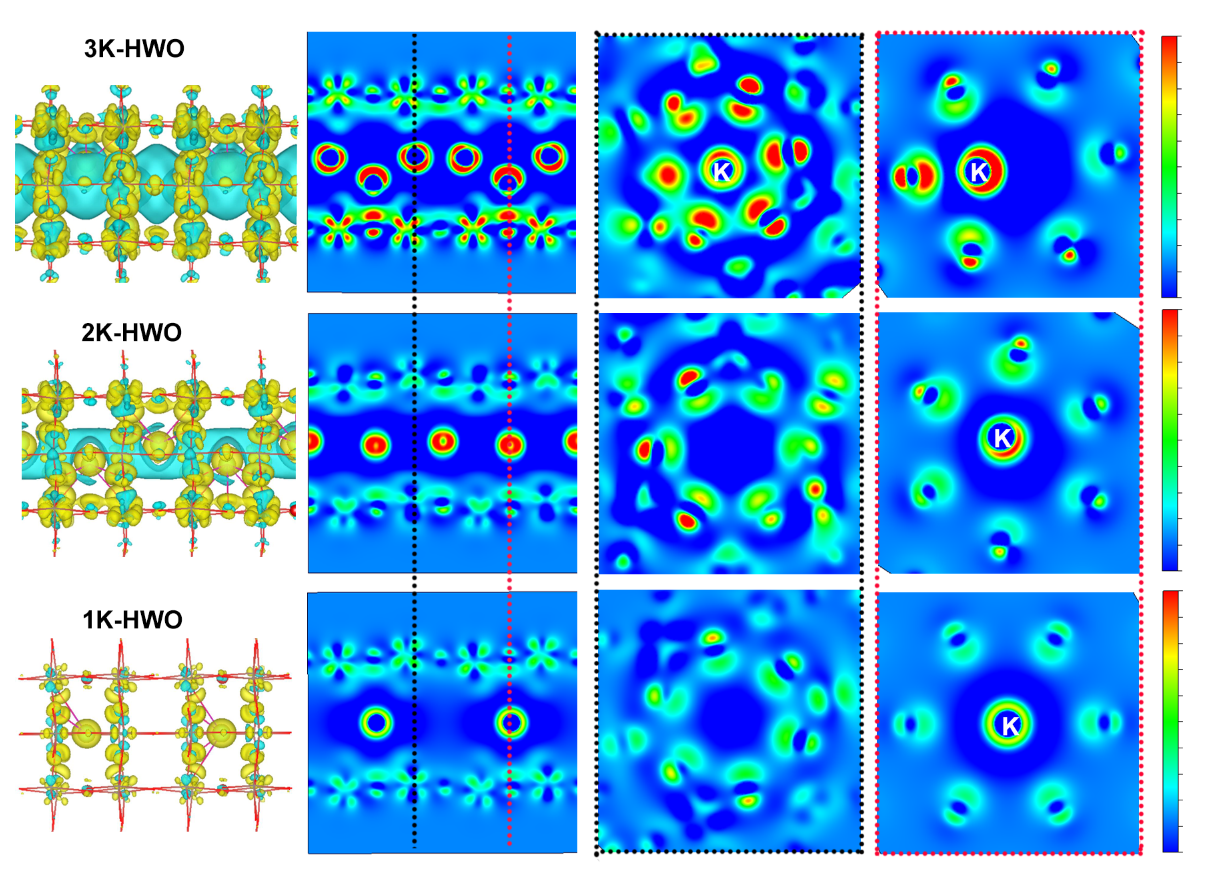


**Figure S12**. The three-dimensional (3D) and two-dimensional (2D) charge density difference maps upon K-atom insertion into the hexagonal WO_3_ tunnels. These patterns show a distinct electronic redistribution of K*_x_*WO_3_ models. In particular, the 3K-HWO system (modeling K_0.37_WO_3_) exhibits significant charge cloud delocalization with pronounced overlap between adjacent K atoms, while the 2K-HWO (modeling K_0.32_WO_3_) and 1K-HWO (modeling K_0.29_WO_3_) systems maintain more localized, isolated charge distributions around individual K sites.


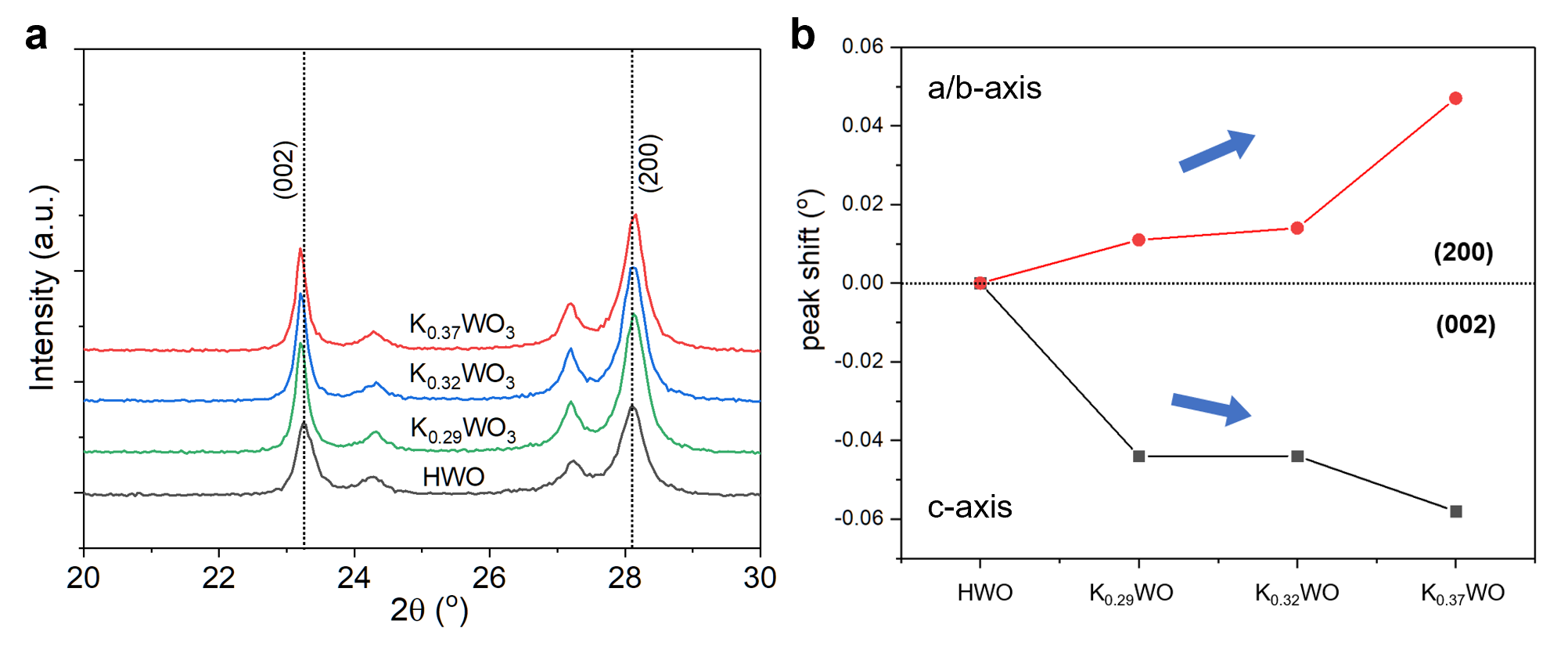


**Figure S13.** (a) Synchrotron X-ray diffraction (SXRD) patterns of HWO and K*_x_*WO_3_ (*x*=0.29, 0.32 and 0.37). (b) The peak shifts of (002) and (200) diffraction peaks of K*_x_*WO_3_ (*x*=0.29, 0.32 and 0.37) compared to HWO. The SXRD patterns of K*_x_*WO_3_ catalysts show that the (002) Bragg reflections shift towards lower angles (up), whilst the (200) Bragg reflections shift towards higher angles (down), indicating an enhanced lattice distortion with the elongation of the c-axis and contraction of the a/b-axis from K_0.29_WO_3_ to K_0.32_WO_3_ and then K_0.37_WO_3_.


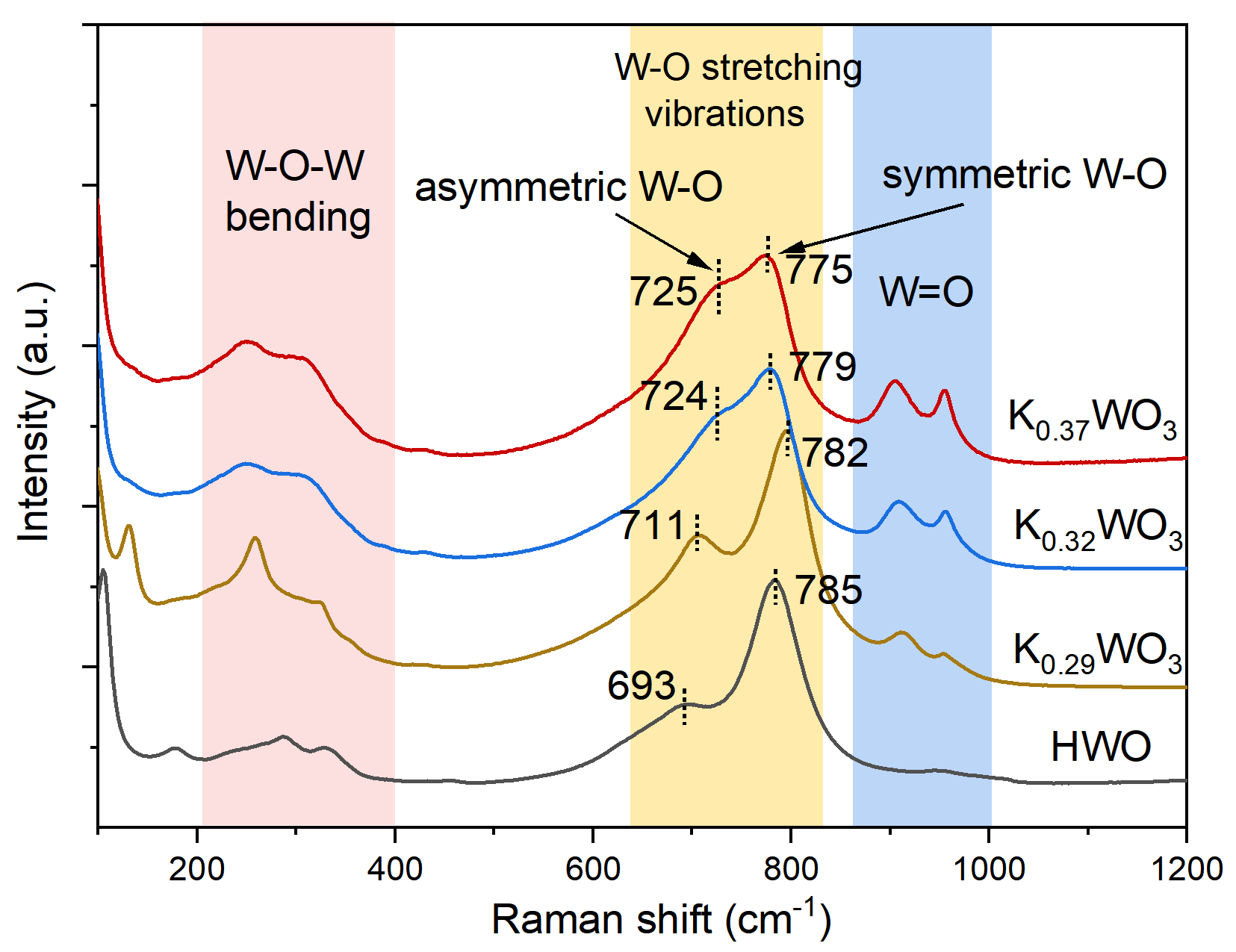


**Figure S14**. Raman spectra of HWO and K*_x_*WO_3_ (*x*=0.29, 0.32 and 0.37) catalysts. The vibration bands below 400 cm^−1^ are attributed to bending vibrations of the WO_6_ octahedra. The bands at 693 cm^−1^ and 785 cm^−1^ are assigned to asymmetric (W-O in a-b plane) and symmetric (W-O along c axis) stretching vibrations of the distorted WO_6_ octahedron, respectively. However, the former shifts to higher wavenumbers, while the latter shifts to the lower wavenumbers in K*_x_*WO_3_ than in HWO with increasing K insertion, indicating the contraction of the W-O bonds along the a/b-axis and the elongation of those along the c-axis, respectively.


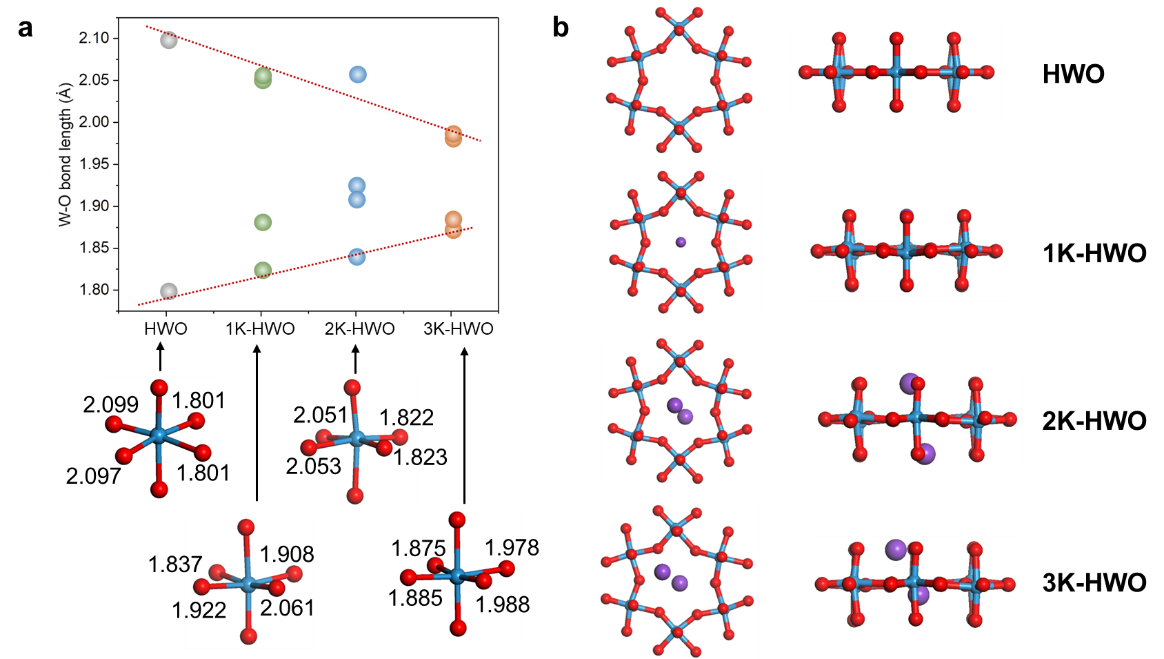


**Figure S15**. (a) The variation tendency of the W-O bond length in the a-b plane for the three models, 1K-HWO, 2K-HWO and 3K-HWO, and (b) the corresponding structures.


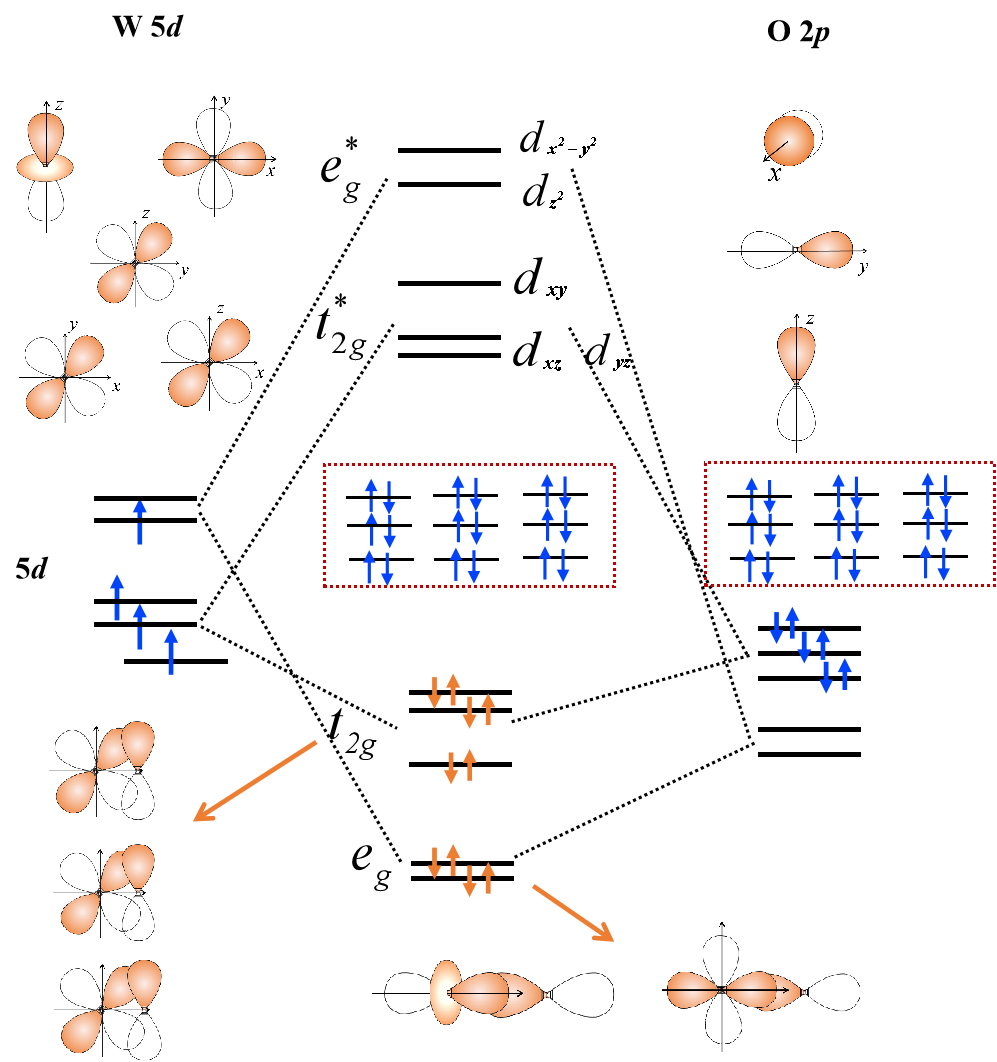


**Figure S16.** Molecular orbital (MO) energy diagram for [WO_6_]^6-^ clusters near the Fermi surface, referring to ref. [9]. ^[9]^


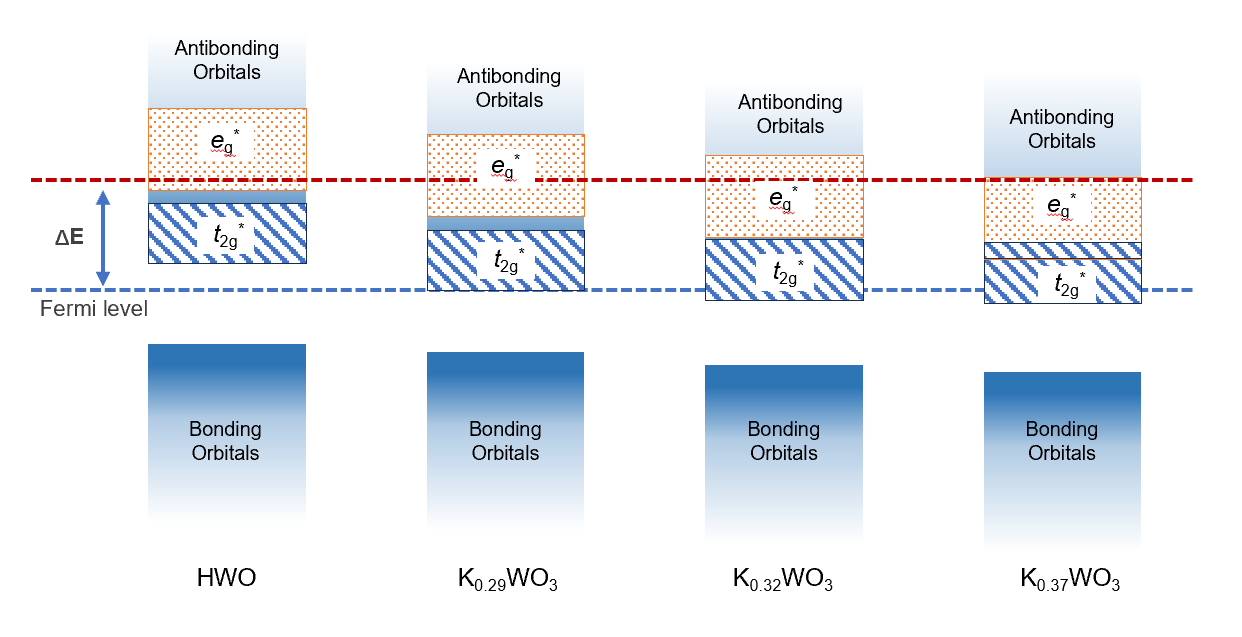


**Figure S17**. The energy band positions of HWO and K*_x_*WO_3_ (*x*=0.29, 0.32 and 0.37) relative to the Fermi level. The red line indicates the highest occupiable energy level for electron transitions under the given characterization conditions. ΔE corresponds to the energy difference between the Fermi level and the highest occupiable energy level.

**Note S3:** In the W *L*_3_-edge XANES spectra, both HWO and K*_x_*WO_3_ samples exhibit two distinct electron transitions, i.e. 2*p*_3/2_→*t*_2g_^*^ and 2*p*_3/2_→*e*_g_^*^ states; however, the peak intensities are varies indicating the probabilities of these transitions vary across different samples. According to the differential XANES spectra with respect to HWO, remarkable enhancements of the *t*_2g_^*^ and *e*_g_^*^peak intensity were observed for K*_x_*WO_3_ compared to HWO, suggesting that it is easier for electrons transition in the system of K*_x_*WO_3_, which should ascribe to the downshift of the antibonding orbitals.

In Figure 3b_1_, the remarkable enhancement of the e_g_^*^ peak for K_0.29_WO_3_ compared to HWO indicates more occupiable e_g_^*^ orbitals remain unoccupied, ascribing to the downshift of the antibonding orbitals, as shown in Figure S17. For K_0.32_WO_3_, the decrease in splitting energy leads to hybridization between the *t*_2g_^*^ and *e*_g_^*^ orbitals, which induces electronic rearrangement. As a result, an increased number of *t*_2g_^*^ and *e*_g_^*^ orbitals become unoccupied, significantly increasing the probability of electron transitions. Furthermore, this pronounced synergistic effect between downward shift of orbital energy levels and orbital hybridization would inherently lead to an increase in transition probability for K_0.37_WO_3_. However, the electron transition probability within the *e*_g_^*^ orbitals is observed to decrease in K_0.37_WO_3_ compared to K_0.32_WO_3_, strongly indicating the occurrence of new electron filling.


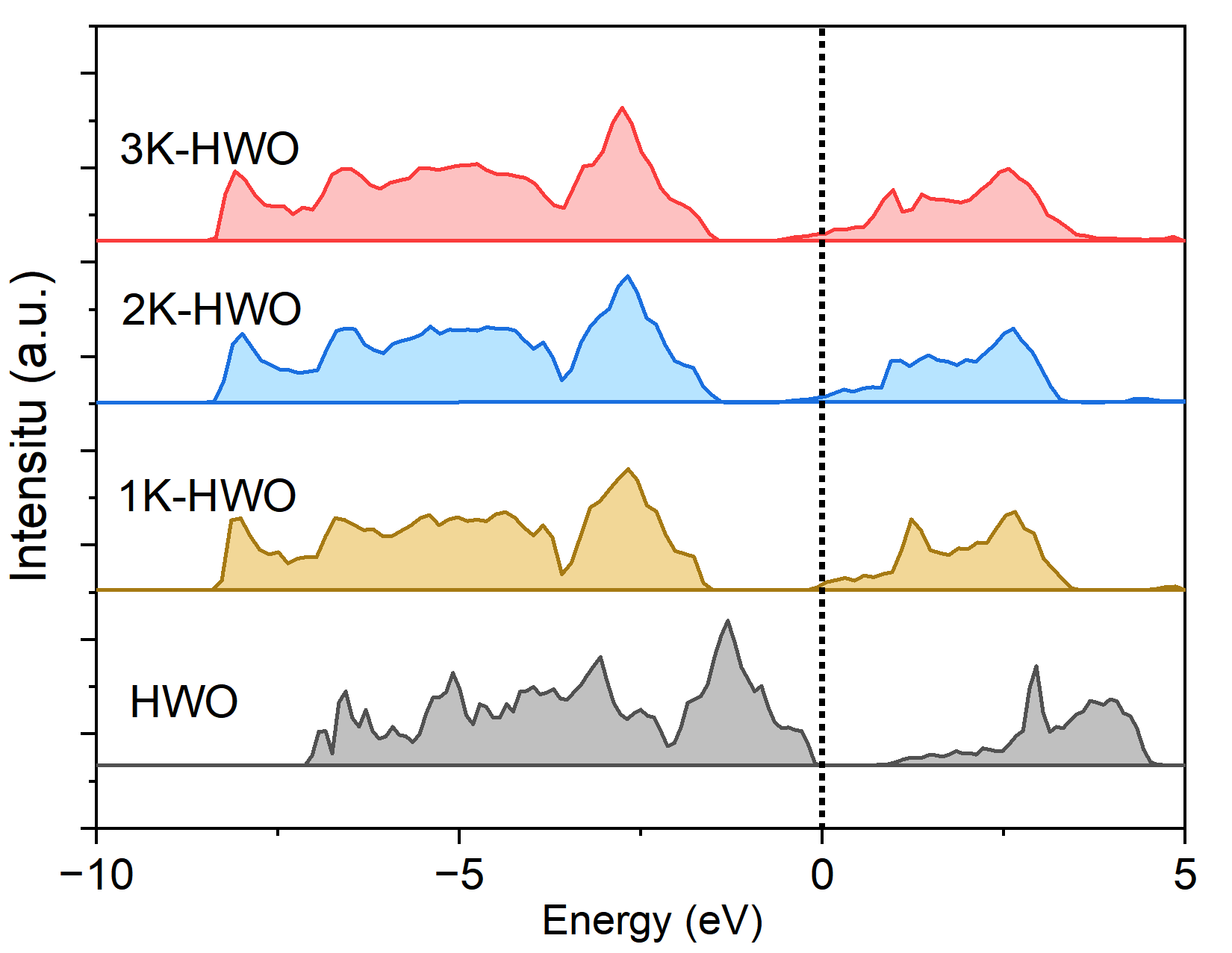


**Figure S18.** Electronic density of states (DOS) of HWO, 1K-HWO, 2K-HWO and 3K-HWO. The Fermi level is located at 0 eV.


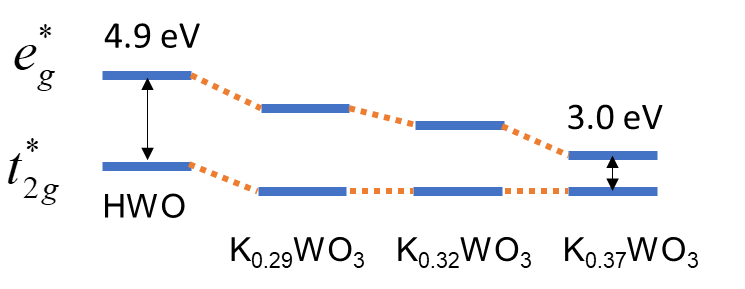


**Figure S19.** The split energy levels for HWO and K*_x_*WO_3_ (*x*=0.29, 0.32 and 0.37).

**
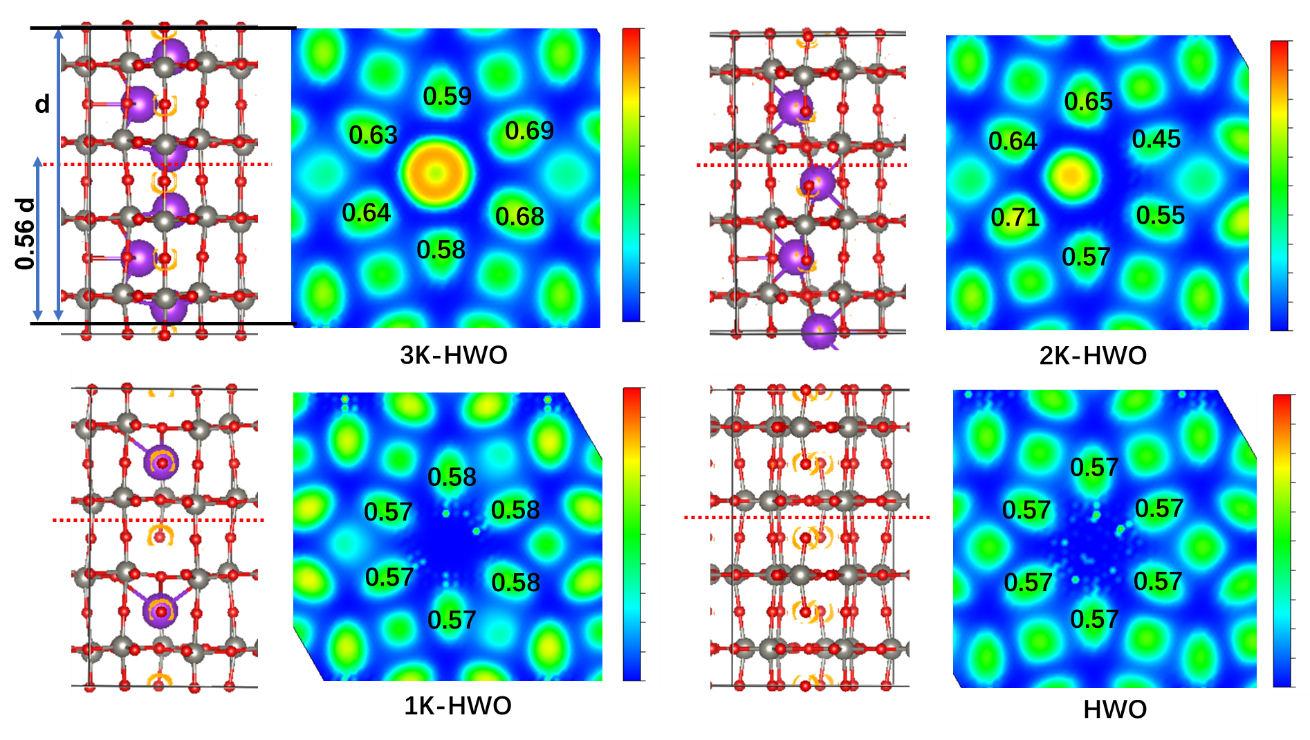
**

**Figure S20**. The geometric structures and color filled 2D maps of electron localization function (ELF) in the range 0.0-1.0. Slice: (001), 0.56*d, where d is the distance of original lattice along c axis.


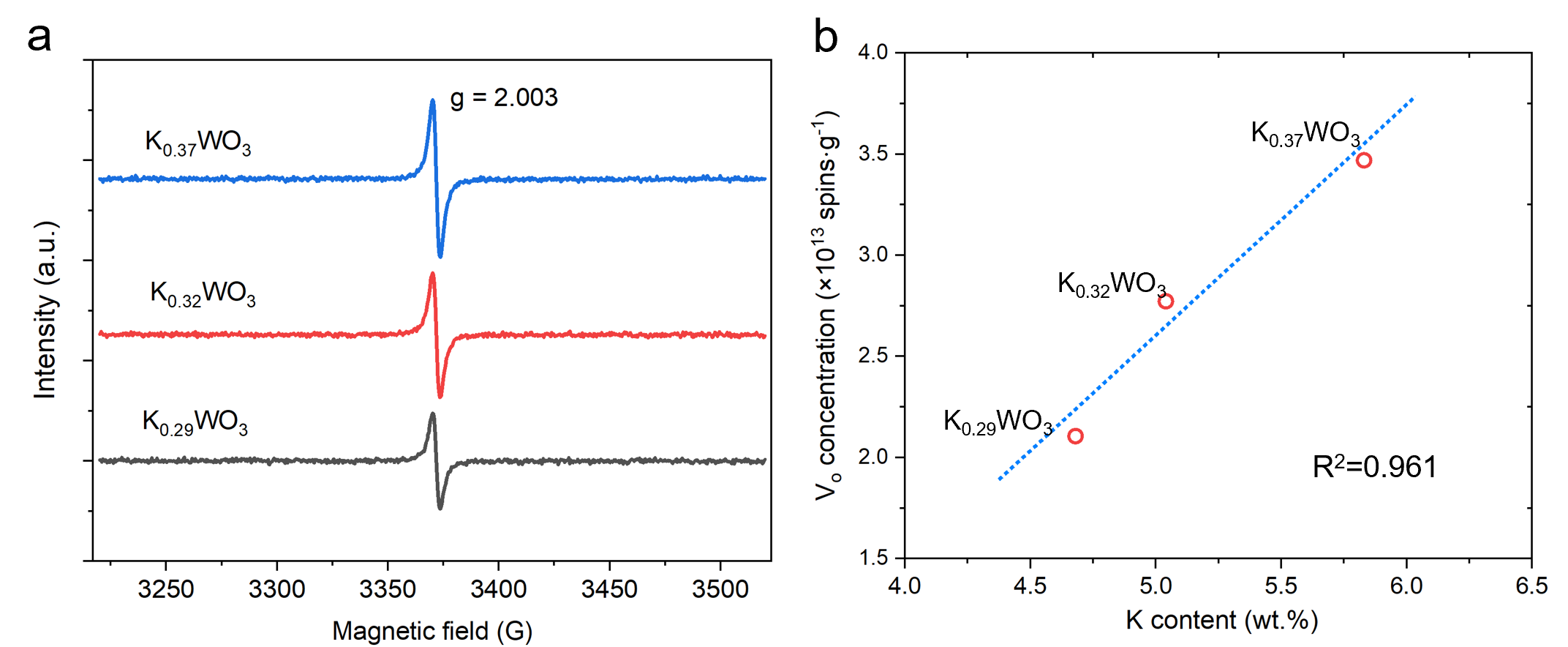


**Figure S21**. (a) EPR spectra of K*_x_*WO_3_ (*x*=0.29, 0.32 and 0.37) catalysts under high vacuum condition. (b) the correlation between V_o_ concentrations and K contents.


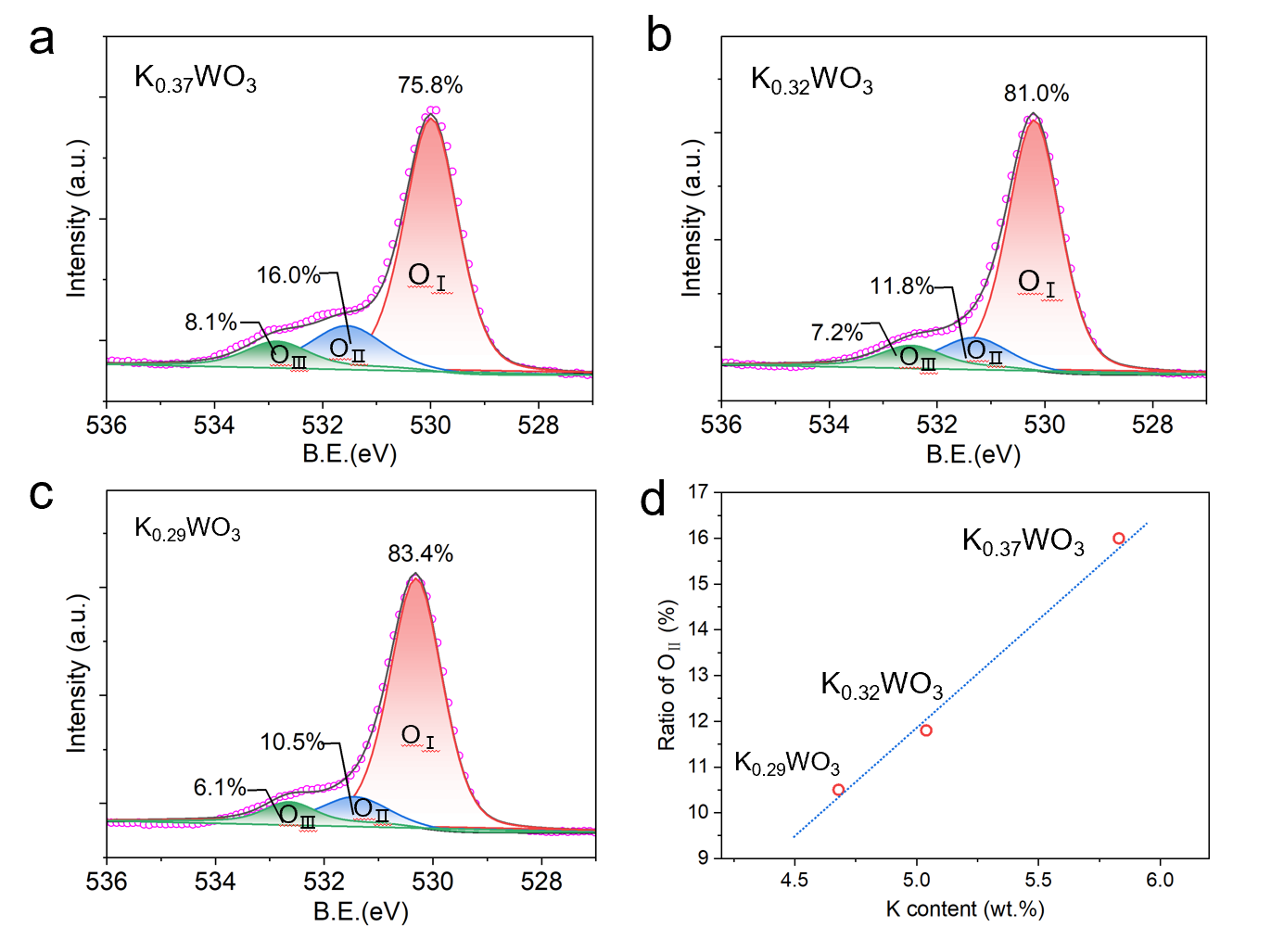


**Figure S22**. (a-c) The peak fitting of O1s XPS results for K*_x_*WO_3_ (*x*=0.29, 0.32 and 0.37), (d) The relationship of the ratio of O_Ⅱ_ and the K content. The three typical XPS peaks at 530.5, 531.6 and 532.8 eV are attributed to the lattice oxygen (O_Ⅰ_), surface-defects oxygen species (O_Ⅱ_) and surface-adsorbed oxygen species (O_Ⅲ_), respectively. The content of oxygen defects (O_Ⅱ_) is related to the concentration of oxygen vacancies in K*_x_*WO_3_ (*x*=0.29, 0.32 and 0.37).


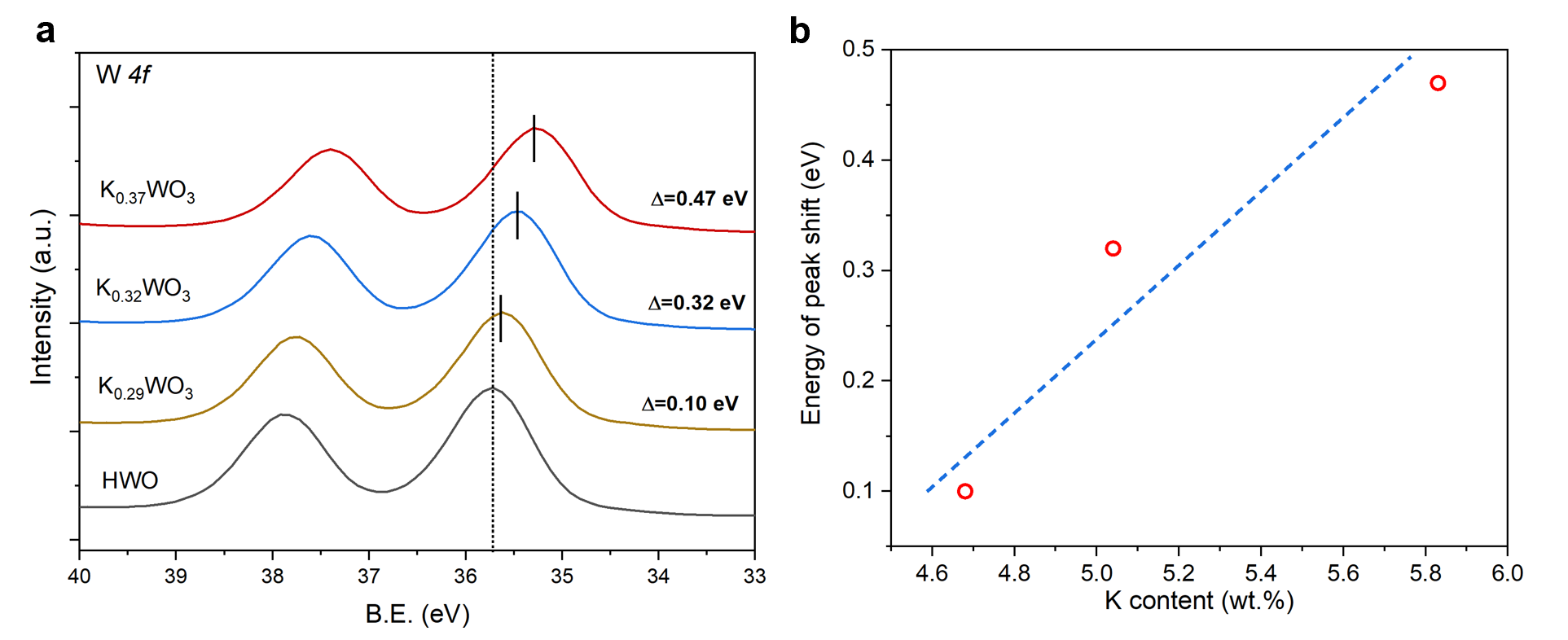


**Figure S23**. (a) The W4f XPS results for HWO and K*_x_*WO_3_ (*x*=0.29, 0.32 and 0.37). (b) the correlation between the energy of peak shift and the K content.


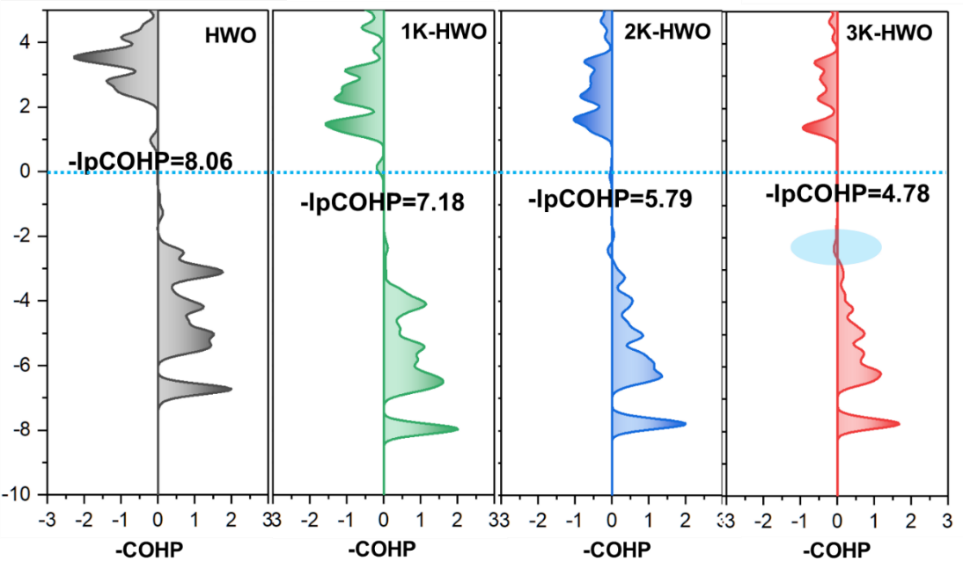


**Figure S24.** The project crystal orbital Hamilton populations (pCOHP) between W atom and O atom. The positive and the negative -COHP represent the bonding contribution and the antibonding contribution, respectively. The integrated values of -pCOHP (-IpCOHP) were used to quantitatively analyze the strength of the interaction between W and O atoms, where the smaller value of -IpCOHP means a weaker interatomic interaction.


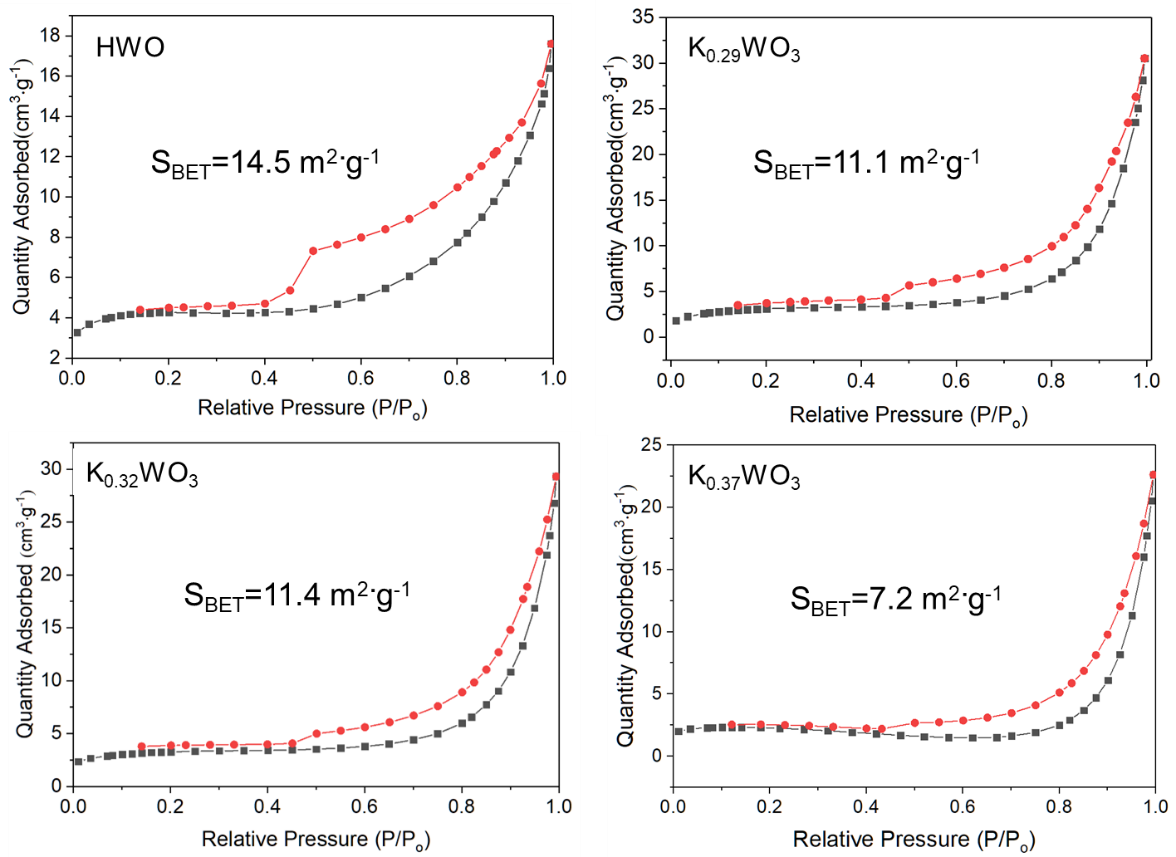


**Figure S25**. N_2_ adsorption/desorption isotherm curves and the corresponding specific surface areas (S_BET_) for K*_x_*WO_3_ (x=0.29, 0.32 and 0.37) and HWO.


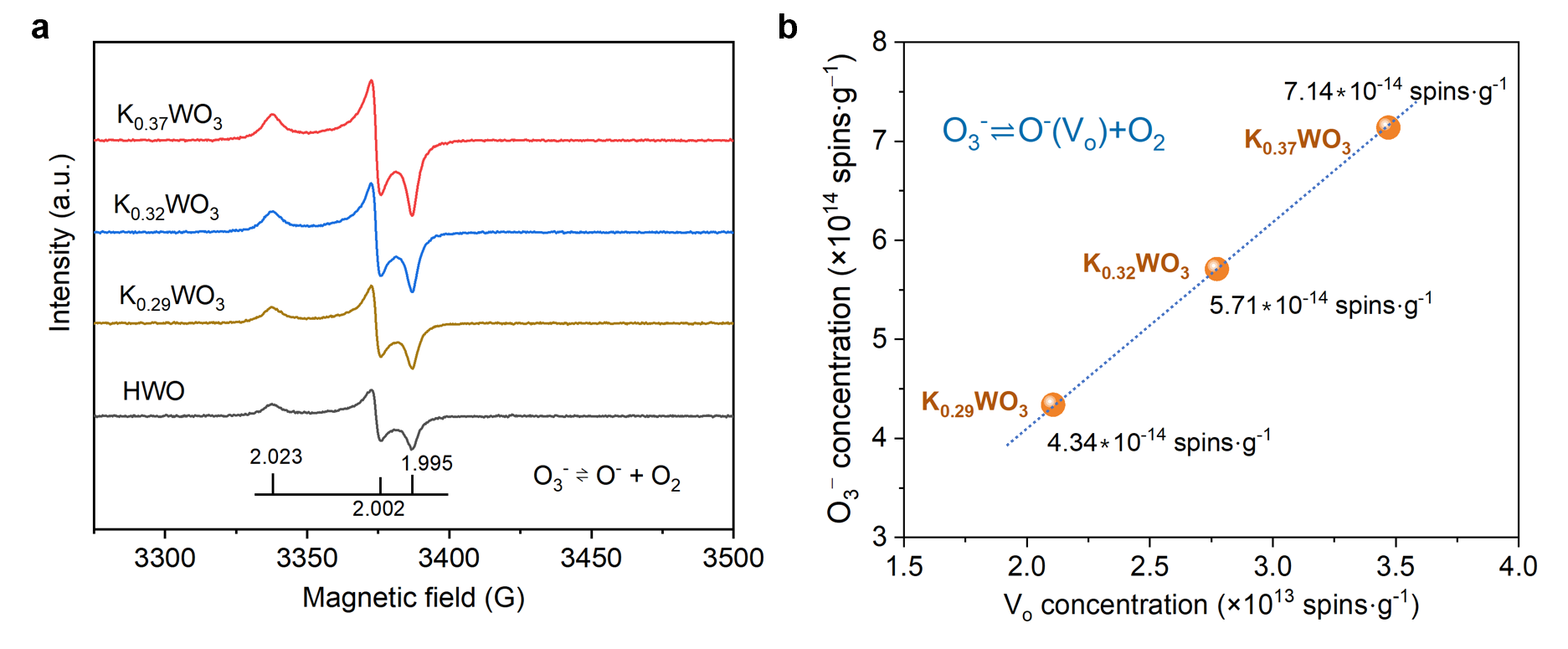


**Figure S26**. (a) Low-temperature EPR spectra of HWO and K*_x_*WO_3_ (*x*=0.29, 0.32 and 0.37) at 77 K in air after high-vacuum treatment (b) the linear correlation between the V_o_ concentration and active oxygen concentration (O_3_^-^).


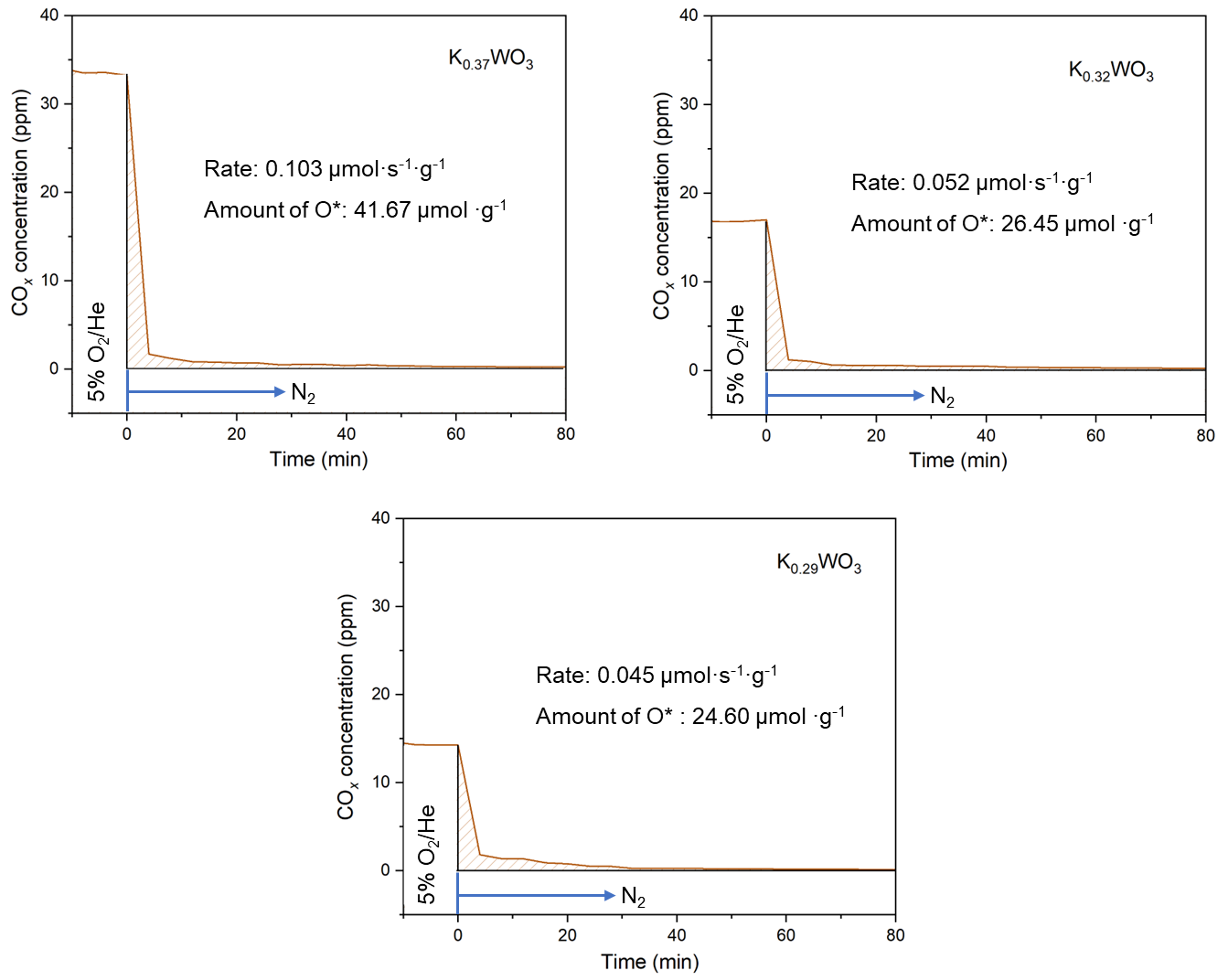


**Figure S27.** CO*_x_* concentrations as a function of time at 390 °C for K*_x_*WO_3_ (*x*=0.29, 0.32 and 0.37) before and after O_2_ is removed from the reactant feed. TOF = reaction rate/amount of O*.


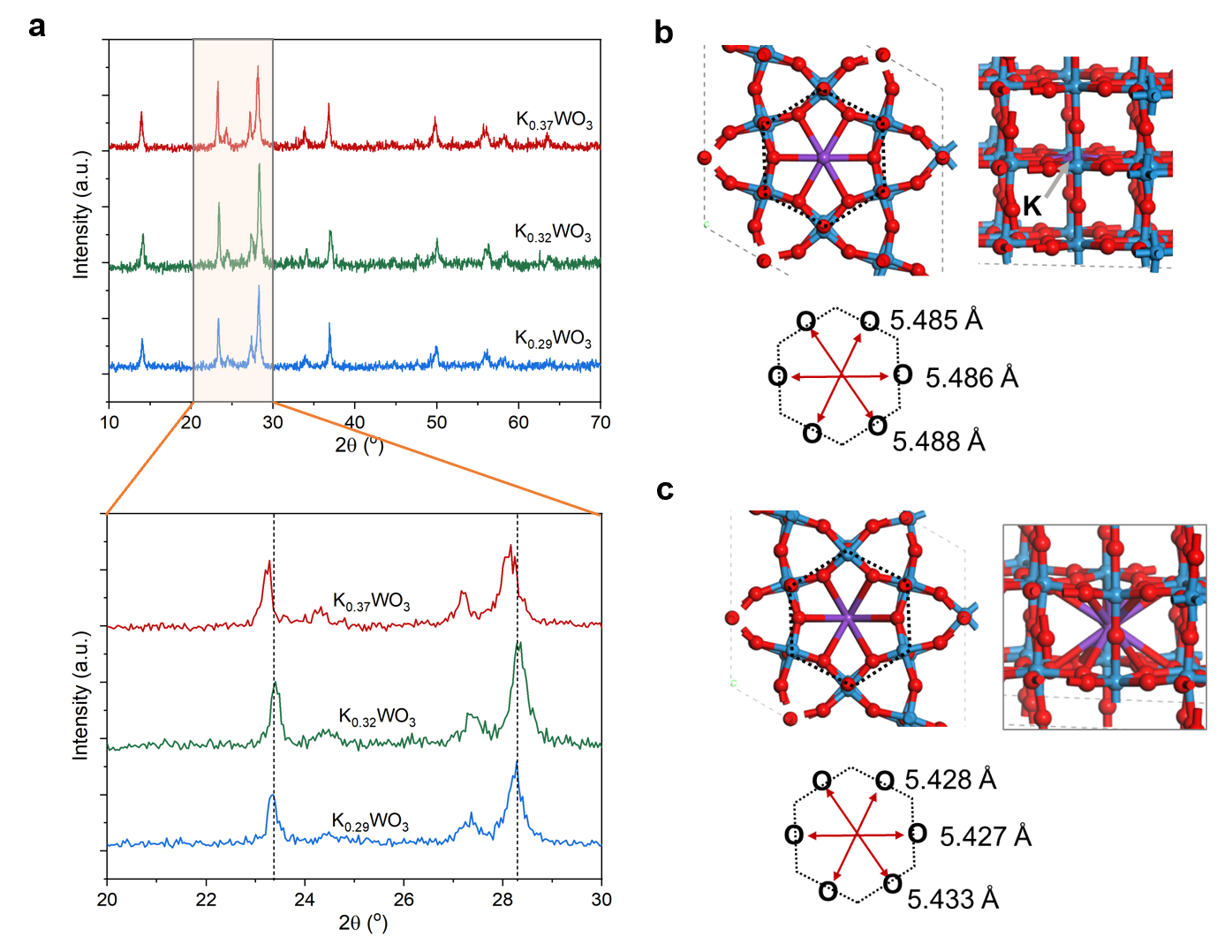


**Figure S28.** (a) XRD patterns of K*_x_*WO_3_ (*x*=0.29, 0.32 and 0.37) after stability tests and corresponding local magnification of the region between 20° and 30°, (b,c) The configurations and the corresponding tunnel size with K_6c_ ions (b) and K_12c_ ions (c).

**Note S4:** In comparison with K_0.29_WO_3_ and K_0.32_WO_3_, the elongation of both *a/b*-axis and *c*-axis of K_0.37_WO_3_ was observed, while the values of the former two were found to be quite similar. The elongation of the c-axis is indicative of enhanced lattice distortion, as evidenced by the changes with increasing K content observed in the fresh sample (Figure S13). However, the slight cell expansion along the a/b-axis for K_0.37_WO_3_ may be attributable to the migration of partial K ions from the K_12c_ site to the K_6c_ site. As shown in Figure S28b and S28c, the tunnel size along *a/b* axis of the configuration with K_6c_ ions (Figure S28b) are larger than that of the configuration with K_12c_ ions (Figure S28c).


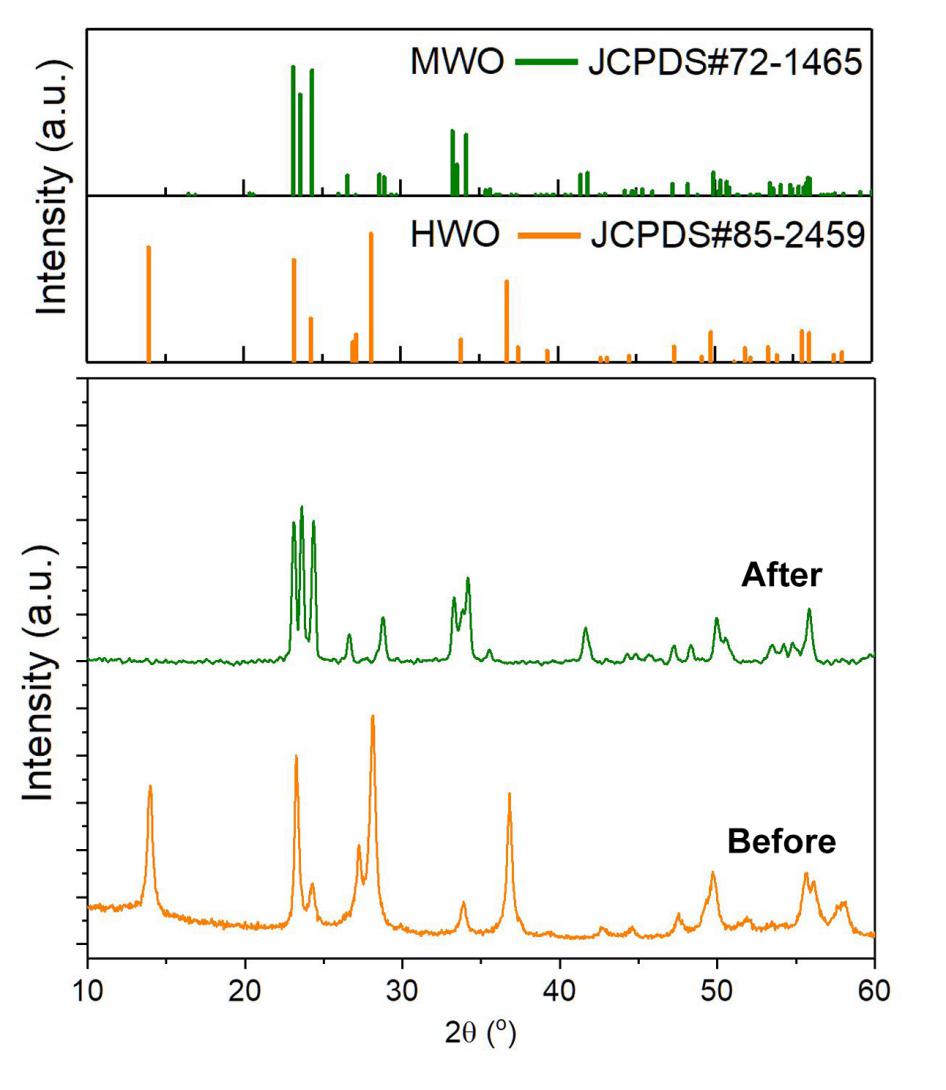


**Figure S29**. XRD patterns of HWO before and after the TPO test. MWO: Monoclinic WO_3_.


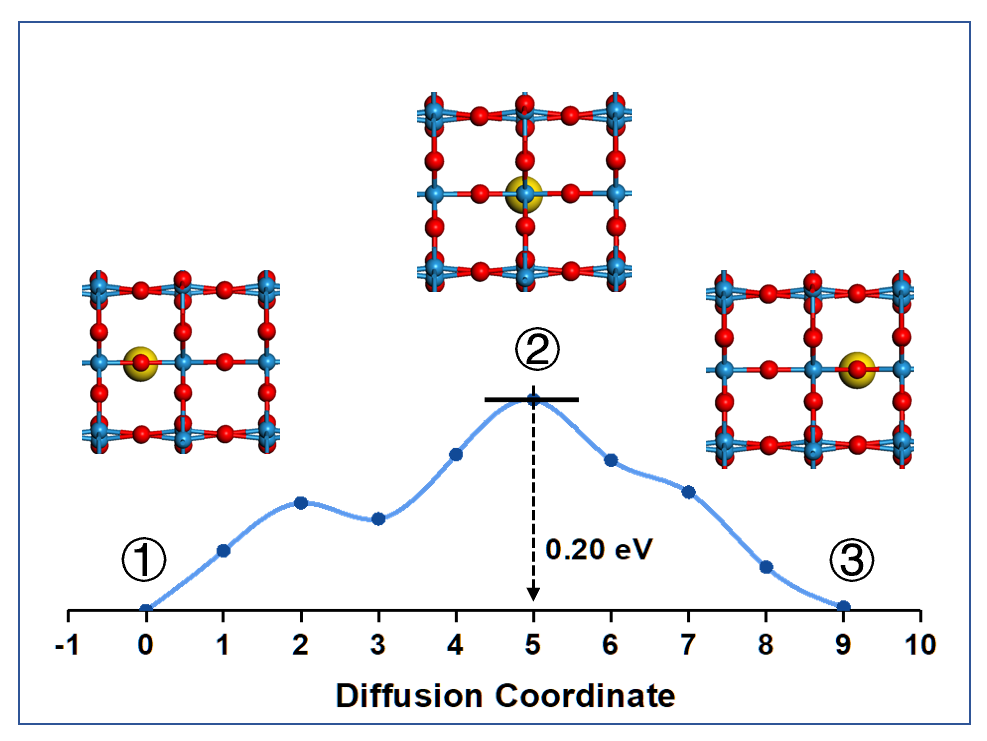


**Figure S30**. The migration trajectories (from site-① to site-② and then site-③) and the corresponding energy profiles of K ion migration within the hexagonal tunnel. Inset: the seria structures of K ions located at several sites in tunnel.

**Supplementary Tables**

**Table S1.** K contents of K*_x_*WO_3_ based on ICP results.

| Samples | K_0.29_WO_3_ | K_0.32_WO_3_ | K_0.37_WO_3_ |
| --- | --- | --- | --- |
| *x* | 0.29 | 0.32 | 0.37 |
| K content | 4.68 wt.% | 5.04 wt.% | 5.83 wt.% |

**Table S2.** Crystallographic data and the Rietveld refinements of HWO and K*_x_*WO_3_ (*x*=0.29, 0.32 and 0.37).

| Samples | HWO | K_0.29_WO_3_ | K_0.32_WO_3_ | K_0.37_WO_3_ |
| --- | --- | --- | --- | --- |
| Chemical formula | WO_3_ | K_0.33_WO_3_ | K_0.33_WO_3_ | K_9_W_24_O_72_ |
| Space group | P 63/mcm | P 63/mcm | P 63/mcm | P1 |
| *a* / Å | 7.3321 | 7.3204 | 7.3194 | 7.2990 |
| *b* / Å | 7.3321 | 7.3204 | 7.3194 | 7.3195 |
| *c* / Å | 7.6434 | 7.6493 | 7.6513 | 7.6553 |
| *V* / Å3 | 355.856 | 354.99 | 355.17 | 355.02 |
| *R*_p_^[b]^ / % | 7.83% | 8.29% | 8.10% | 8.23% |
| *R*_wp_^[b]^ / % | 9.8% | 10.7% | 10.4% | 10.95% |

**Table S3**. The cell parameters of K*_x_*WO_3_ after stability tests.

| Samples | a/Å | b/Å | c/Å |
| --- | --- | --- | --- |
| K_0.29_WO_3_-A | 7.289 | 7.289 | 7.637 |
| K_0.32_WO_3_-A | 7.281 | 7.281 | 7.633 |
| K_0.37_WO_3_-A | 7.318 | 7.318 | 7.642 |

**Table S4.** Comparison of the soot combustion of our synthesized K_0.37_WO_3_ catalyst with relevant catalysts in the literature.

| Samples | Reaction Conditions | *T*_max_*/T*_50_/℃ | References |
| --- | --- | --- | --- |
| Nepheline (KNa_3_Al_4_Si_4_O_16_) | 10% O_2_/He, Loose contact | 540 | 1^[10]^ |
| Kalsilite (K_4_Al_4_Si_4_O_16_) | 10% O_2_/He, Loose contact | 600 | 1 |
| Kaliophilite (K_4_Al_4_Si_4_O_16_) | 10% O_2_/He, Loose contact | 580 | 1 |
| Potassium disilicate glasses (K_2_Si_2_O_5_) | 10%O_2_/5%CO_2_/N_2_/3%H_2_O,  TGA | 375 | 2 ^[11]^ |
| Birnessite (K_0.56_MnO_2+x_) | 0.05%NO*_x_*/5%O_2_/N_2_, Loose contact | 450 | 3 ^[12]^ |
| Cryptomelane(K_0.12_MnO_2+x_) | 0.05%NO*_x_*/5%O_2_/N_2_, Loose contact | 480 | 3 |
| Turbostratic Birnessite  (K_0.21_MnO_2+x_) | 0.05%NO*_x_*/5%O_2_/N_2_, Loose contact | 450 | 4 ^[13]^ |
| Cryptomelane K_1.7_Mn_8_O_16_ | 5% O_2_/He, Tight contact | 407 | 5 ^[14]^ |
| Hollandite K_1.2_Ti_8_O_16_ | 5% O_2_/He, Tight contact | 460 | 5 |
| Birnessite K_2_Mn_4_O_8_ | 10%O_2_/0.2%NO/10%H_2_O/Ar, Loose contact | 316 | 6 ^[15]^ |
| Cryptomelane K_2-x_Mn_8_O_16_ | 10%O_2_/0.2%NO/10%H_2_O/Ar, Loose contact | 332 | 6 |
| Hexagonal K_0.37_WO_3_ | 5% O_2_/He, Tight contact | 504 | This work |

**Note S5:** As shown in Table S4, this table specifically highlights two important types of confined potassium catalysts for catalytic soot combustion.

In contrast to the catalysts containing K^+^ ions embedded in a silicate matrix (**Type 1**, Table S4 line 1-4) in Ref 1 and Ref 2, the K_0.37_WO_3_ catalyst exhibit higher activity with lower *T*_50_ under similar test conditions. Notable, the outward migration of K^+^ ions from the bulk to the surface of the glass catalyst enables sustained surface potassium availability for long-term catalytic soot combustion. However, our study employs a tailored K–O coordination environment to significantly enhance the stability of confined K ions. Furthermore, the constructed K–K interactions with forming K^δ+^ (0<δ<1) species promote catalytic activity by activating lattice oxygen species.

With respect to the catalysts with K^+^ ions within a MnO_2_ matrix in the relevant literature (**Type 2**, Table S4 line 5-11) in Ref 3-6, the K_0.37_WO_3_ catalyst shows a lower activity with higher *T*_50_. However, the alkali promoter effect, general resulting from the forming of abundant oxygen vacancy, is largely overshadowed by the high inherent redox activity of α-MnO_2_. Using hexagonal tungsten trioxide (W-O is rather inert) as the host, the present study is the first to observe and systematically investigate the direct electron donation effect of K^δ+^ (0<δ<1) ions, which not only facilitate the formation of oxygen vacancies but more importantly also directly donate electrons to [WO_6_] motif antibonding orbitals, thereby triggering the lattice oxygen activation.

**Supplementary References**

[1] Zhang, Z.; Han, D.; Wei, S.; Zhang, Y. Determination of active site densities and mechanisms for soot combustion with O_2_ on Fe-doped CeO_2_ mixed oxides. *J. Catal.* **2010,** *276* (1), 16.

[2] Dronskowski, R.; Bloechl, P. E. Crystal orbital Hamilton populations (COHP): energy-resolved visualization of chemical bonding in solids based on density-functional calculations. *J. Phys. Chem.* **1993,** *97* (33), 8617.

[3] Maintz, S.; Deringer, V. L.; Tchougréeff, A. L.; Dronskowski, R. LOBSTER: A tool to extract chemical bonding from plane-wave based DFT. *J. Comput. Chem.* **2016,** *37* (11), 1030.

[4] Lee, J.-S.; Liu, H.-C.; Peng, G.-D.; Tseng, Y. Facile synthesis and structure characterization of hexagonal tungsten bronzes crystals. *J. Cryst. Growth.* **2017,** *465*, 27.

[5] Zheng, Z.; Yan, B.; Zhang, J.; You, Y.; Lim, C. T.; Shen, Z.; Yu, T. Potassium tungsten bronze nanowires: polarized micro-Raman scattering of individual nanowires and electron field emission from nanowire films. *Adv. Mater.* **2008,** *20* (2), 352.

[6] Li, L.; Jiang, F.; Tu, F.; Jia, S.; Gao, Y.; Wang, J. Atomic-scale study of cation ordering in potassium tungsten bronze nanosheets. *Adv. Sci.* **2017,** *4* (9), 1600537.

[7] Guo, C.; Yin, S.; Huang, L.; Sato, T. Synthesis of one-dimensional potassium tungsten bronze with excellent near-infrared absorption property. *ACS Appl. Mater. Interfaces.* **2011,** *3* (7), 2794.

[8] Hou, J.; Liu, L.; Li, Y.; Mao, M.; Lv, H.; Zhao, X. Tuning the K^+^ concentration in the tunnel of OMS-2 nanorods leads to a significant enhancement of the catalytic activity for benzene oxidation. *Environ. Sci. Technol.* **2013,** *47* (23), 13730.

[9] Yoshio, S.; Okada, M.; Adachi, K. Destabilization of pseudo-Jahn–Teller distortion in cesium-doped hexagonal tungsten bronzes. *J. Appl. Phys.* **2018,** *124* (6), 063109.

[10] Kimura, R.; Wakabayashi, J.; Elangovan, S. P.; Ogura, M.; Okubo, T. Nepheline from K_2_CO_3_/nanosized sodalite as a prospective candidate for diesel soot combustion. *J. Am. Chem. Soc.* **2008,** *13012844-12845*.

[11) Su, C.; McGinn, P. J. The effect of Ca^2+^ and Al^3+^ additions on the stability of potassium disilicate glass as a soot oxidation catalyst. *Appl. Catal. B-Environ.* **2013,** *138-139*, 70.

[12] Atribak, I.; Bueno-López, A.; García-García, A.; Navarro, P.; Frías, D.; Montes, M. Catalytic activity for soot combustion of birnessite and cryptomelane. *Appl. Catal. B-Environ.* **2010,** *93* (3-4), 267.

[13] Becerra, M. E.; Arias, N. P.; Giraldo, O. H.; López Suárez, F. E.; Illán Gómez, M. J.; Bueno López, A. Soot combustion manganese catalysts prepared by thermal decomposition of KMnO_4_. *Appl. Catal. B-Environ.* **2011,** *102* (1-2), 260.

[14] Liu, T.; Li, Q.; Xin, Y.; Zhang, Z.; Tang, X.; Zheng, L.; Gao, P.-X. Quasi free K cations confined in hollandite-type tunnels for catalytic solid (catalyst)-solid (reactant) oxidation reactions. *Appl. Catal. B-Environ.* **2018,** *232*, 108.

[15] Yu, D.; Ren, Y.; Yu, X.; Fan, X.; Wang, L.; Wang, R.; Zhao, Z.; Cheng, K.; Chen, Y.; Sojka, Z.et al. Facile synthesis of birnessite-type K_2_Mn_4_O_8_ and cryptomelane-type K_2-_*_x_*Mn_8_O_16_ catalysts and their excellent catalytic performance for soot combustion with high resistance to H_2_O and SO_2_. *Appl. Catal. B-Environ.* **2021,** *285*.
